# Fingerprinting Electronic Molecular Complexes in Liquid

**DOI:** 10.1038/srep19009

**Published:** 2016-01-08

**Authors:** Peter Nirmalraj, Andrea La Rosa, Damien Thompson, Marilyne Sousa, Nazario Martin, Bernd Gotsmann, Heike Riel

**Affiliations:** 1 IBM Research – Zurich, Säumerstrasse 4, CH- 8803 Rüschlikon, Switzerland; 2 Departmento de Quimica Organica, Facultad de Quimica, Universidad Complutense de Madrid, E-28040, Madrid, Spain; 3 Department of Physics and Energy, University of Limerick, Ireland; 4 Materials and Surface Science Institute, University of Limerick, Ireland

## Abstract

Predicting the electronic framework of an organic molecule under practical conditions is essential if the molecules are to be wired in a realistic circuit. This demands a clear description of the molecular energy levels and dynamics as it adapts to the feedback from its evolving chemical environment and the surface topology. Here, we address this issue by monitoring in real-time the structural stability and intrinsic molecular resonance states of fullerene (C_60_)-based hybrid molecules in the presence of the solvent. Energetic levels of C_60_ hybrids are resolved by *in situ* scanning tunnelling spectroscopy with an energy resolution in the order of 0.1 eV at room-temperature. An ultra-thin organic spacer layer serves to limit contact metal-molecule energy overlap. The measured molecular conductance gap spread is statistically benchmarked against first principles electronic structure calculations and used to quantify the diversity in electronic species within a standard population of molecules. These findings provide important progress towards understanding conduction mechanisms at a single-molecular level and in serving as useful guidelines for rational design of robust nanoscale devices based on functional organic molecules.

To decipher the conductance spectrum of molecules requires an in-depth knowledge of molecular binding geometry, intermolecular interactions and access to unperturbed molecular energy levels. Bonding geometries in metal-molecule-metal junctions^1^,^2^,^3^,^4^,^5^ and elementary conformational shifts in the molecular structure^6^,^7^,^8^,^9^ have been widely discussed as the source of variations in single-molecule conductance. The structural stability of the contact metal^10^, local chemical potential of the molecular environment^11^,^12^, hydration effects^13^, trapped charges at the metal-organic interface^6^,^14^, temperature^15^, intermolecular interactions^16^ and chemical functionality^17^ are other factors that can contribute to the spread in values of molecular quantum conductance (G_0_) and tunneling attenuation factor (β) values. These arguments are valid in the case of relatively simple and short-length molecules^18^ wired between metal electrodes. However, the root cause for differences in conductance in the case of more complex molecular architectures where there are additional degrees of freedom remains to be fully accounted for at a single-molecular level. For example, assemblies involving linear molecular moieties chemically linked to anchor groups that serve as extended electrodes, which are prototype molecular electronics components. Fullerenes (C_60_) have been actively explored as molecular anchor groups^19^ owing to their excellent bonding with metals and low contact resistance. Recent experiments from mechanically controllable break-junctions (MCBJ)^20^,^21^ to scanning tunnelling microscopy (STM)^22^,^23^ and density functional theory (DFT) calculations^24^,^25^ of C_60_ based complexes have helped understand the charge transport process in these systems. However, the experimental and theoretical studies on C_60_ dimers (C_60_—linker—bridge—linker—C_60_) report on structurally stable molecules and do not take into account the possibility of mixed electronic species, which has limited the interpretation of measured conductance values and charge propagation modes.

## Resolving Molecular Complex Structure in Liquids

**Figure 1:**
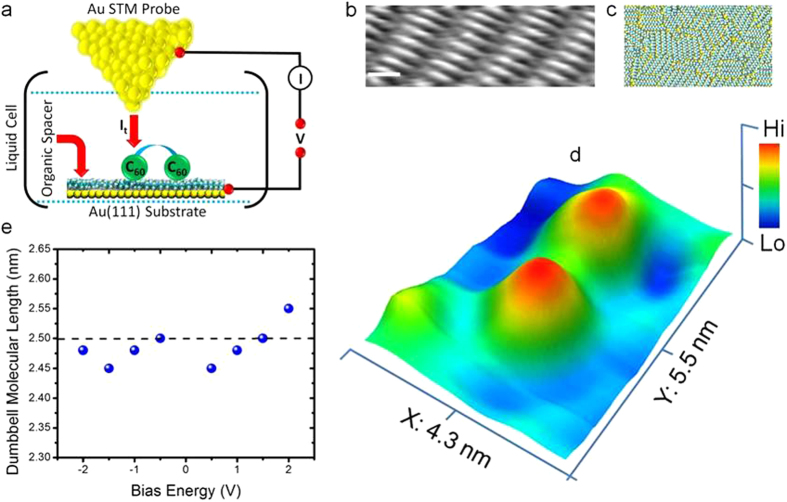
Measuring single-molecular structure in liquids. (**a)** Schematic detail of the *in situ* STM/STS design. (**b)** Constant-current STM image of an ordered *n-*C_14_H_30_ molecular spacer layer on Au(111) (tunnelling parameters : I = 400 pA, V = −1.3 V scale bar: 1 nm). (**c)** Atom-scale computed structure of the early stages of *n*-C_14_H_30_ assembly on Au(111), formed after twenty nanoseconds of equilibrated room temperature molecular dynamics in *n*-C_14_H_30_ solvent. Solvent molecules have been excluded for clarity. Atoms are shown as space-filling spheres, and each *n*-C_14_H_30_ has a molecular length of ~15 Å. The full simulation cell is described in Supplementary Section S9, and contains 600 *n*-C_14_H_30_ molecules adsorbed on a 33 nm × 13 nm slab of Au(111) immersed in a cell of 3750 bulk *n*-C_14_H_30_ molecules. (**d)** High-resolution *in situ* STM image of a regular C_60_ dimer molecule with a dumbbell shaped architecture (tunnelling parameters: I = 25 pA, V = 0.3 V). (**e**) Molecular length (center-to-center) analysis as a function of the applied bias energy.

Previously, the structure of molecules and metal adatoms within an organic matrix has been visualized^26^,^27^,^28^,^29^,^30^,^31^, the dynamics of molecular adsorbates recorded^32^,^33^ and the thermodynamic equilibrium of complex networks previously probed^34^ at the liquid-solid interface. Here, we resolve the electronic structure of a single isolated molecule in a liquid environment at room-temperature with high-spatial, temporal and energy sensitivity using our *in situ* (within the liquid medium) scanning tunnelling microscope (*in situ* STM)/spectroscope (*in situ* STS) setup^35^,^36^ (Fig. 1a). The entire experimental procedure was conducted in a noise-free environment^37^. The justification for performing such nanoscopic measurements on C_60_ dimers in liquids is mainly because this class of molecular complexes is not compatible with vapor-phase deposition and by performing such measurements in a wide range of solvents, the role of the encompassing solvent on the molecular electronic structure and molecular structural stability can be verified. From real-space and time-elapsed STM studies we observe that in addition to the expected regular dimers there exists a small population of new molecular species of individual C_60_ components with a strikingly different electronic structure (verified using STS) in comparison to the regular dimer counterparts. This observation of a non-homogenous distribution of molecular electronic structures can explain the spread in the previously reported conductance values of fullerene anchor based molecular complexes^21^,^23^ and other large molecular structures involving similar geometrical design^38^, where the molecules are deposited from liquid-phase.

Regular C_60_ dimer molecules (fluorene-spaced molecular wires with C_60_ anchor units, chemical structure and synthesis are shown in Supplementary Section S1) solubilised in *n*-tetradecane solvent were deposited on an alkane-protected Au(111) surface by controlled deposition inside a liquid-cell. Previously, we demonstrated the application of an alkane (*n*-C_30_H_62_) molecular layer to electronically decouple adsorbed low-dimensional organics from the underlying metal surface^36^. In the current work we employ an *n*-C_14_H_30_ spacer layer (for fabrication see Supplementary Section S2) with comparable electrochemical properties (ε = 2.0 and conductance bandgap: ~14 eV). The organic electronic decoupling platforms can be readily engineered by the self-assembly of alkane molecules into a compact layer on Au(111).

Figure 1b shows an STM image obtained in constant-current mode of an ordered monolayer of *n*-C_14_H_30_ adsorbed on Au(111). The mean molecular length and intermolecular spacing between the side by side packed *n*-C_14_H_30_ units is (1.5 ± 0.1) nm and ~0.4 nm, respectively, in good agreement with previous reports^39^. Separate experiments in which the C_60_ dimers (solublised in *n*-tetradecane solvent) were directly deposited onto a clean Au(111) surface also resulted in partial ordering of the alkanes. However, the alkane layer was not continuous over large sections (verified using *in situ* STM) as the alkanes enter into a direct energetic competition with the C_60_ dimers for adsorption onto the Au(111) surface. This justifies the deposition of C_60_ dimers onto a pre-formed rigid and homogeneous spacer layer (confirmed using *ex-situ* ellipsometry, see Fig. 3 in Supplementary Section S2). The choice of *n*-tetradecane as the solvent stems from its electrochemical inertness, low-volatility which ensures stable *in situ* STM imaging and its ability to solvate C_60_ derivatives. Atom-scale modelling was performed to quantify the spacer layer interactions with the gold surface. Based on computed structures of *n*-C_14_H_30_ on Au (111) (Fig. 1c, details are in supplementary section S9), an intermolecular packing energy of (−0.7 ± 0.1) eV/molecule (within a computed monolayer density of 1.9 × 10^−10^ molecules/cm^2^) and molecule-gold adsorption energy of (−2.3 ± 0.3) eV/molecule is calculated, indicating a strongly adsorbed and tightly packed monolayer. The regular C_60_ dimer is well-resolved from the high-resolution *in situ* STM image (Fig. 1d) recorded under low-bias conditions (directly after the liquid-phase deposition). The molecular length does not exhibit a strong dependence on the bias energy (Fig. 1e) when measured at low-bias (−2 V, 2.48 nm) and at higher-biases (+2 V, 2.55 nm) and yields a near-constant mean molecular length of (2.5 ± 0.05, center-to-center distance) nm averaged over ~120 C_60_ dimers. This confirms that the regular C_60_ dimers linked through the chemical bridge remain intact under a wide-working energy range (−2 to +2 V). The mean molecular length value obtained from the *in situ* STM data is close to previous STM measurements taken in dry conditions (solvent evaporated after molecular deposition) on related C_60_ dimers on Au (111)^23^.

## Determination of Single-Molecular Energy Spectrum

**Figure 2:**
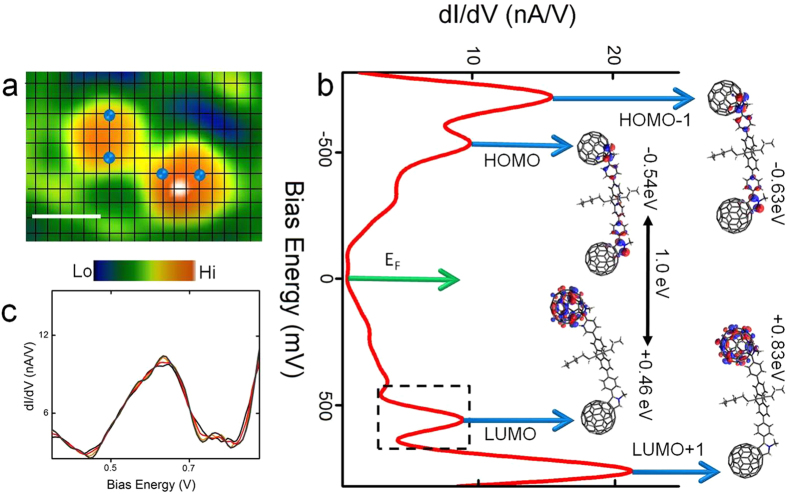
Electronic structure of regular C_60_ dimers. (**a**) *In situ* STM image of a regular C_60_ dimer on which the individual spectral curves were recorded at the locations indicated by the blue spheres on the grid (tunnelling parameters: I = 5 pA, V = 0.2 V, scale bar: 2 nm). (**b**) d*I*/d*V* spectra (averaged over spectra recorded at four points as shown in panel a) (Set point: I = 120 pA, V_s_ = 0.6 V). DFT calculated frontier molecular orbitals are indicated by the blue arrows. (**c**) Close-up of the LUMO peak (as indicated by the dashed black box in panel b) for all the four individually acquired spectra.

We first examine the molecular energy levels of regular C_60_ dimers adsorbed on *n*-C_14_H_30_ spacer layer coated Au(111) in a liquid medium. The local electronic interaction between a monolayer of *n*-C_14_H_30_ and Au(111) in liquids has been previously discussed^40^ and *n*-tetradecane has been demonstrated to serve as a reliable liquid sheath model system in which tunnelling spectroscopic measurements can be performed on organic complexes without any electrical interference from the encompassing liquid^30^,^40^,^41^. A regular C_60_ dimer is located (Fig. 2a) and its dimensions and stability over time is verified by continuous STM imaging. On confirming molecular stability, the STM probe is then positioned at a specific point on a C_60_ lobe and the feedback loop is opened at a fixed height above the molecule and the voltage is swept (−1 V to +1 V) while the current is recorded. The STM tip drift rate is ~1 nm/min in *n*-tetradecane solvent, with the feedback loop re-initiated between acquiring spectral data to ensure that the structure and position of the molecule remains unchanged after each spectroscopic reading at the different points marked as blue spheres in Fig. 2a. The STS spectra were acquired on the molecular species using several Au tips prepared using identical protocols to check for reproducibility. Although, there were variations in the spectral intensities, the overall line shape and peak positions did not alter drastically and the minute differences have been quantified with experimental error rates, quantified in the energy gap distributions. The structure of the molecule and position of the tip was constantly verified before and after acquisition of the spectroscopic readings (see Supplementary Section S6 for details of spectroscopic measurement protocols). The electrochemical inertness and high density of the *n*-tetradecane solvent medium (see Table 2 Supplementary Section S3 for solvent properties) further ensures consistent tunnelling conditions by protecting the tunnel gap against moisture buildup which is known to induce barrier height fluctuations at the liquid-solid electrical interface^42^. Molecular dynamics calculations (details are in Supplementary Section S9) show strong adhesion of the molecules to the surface in a mixture of on-gold and on-spacer binding modes with low computed molecular motions in the *n*-tetradecane medium.

Figure 2b is a dI/dV spectral curve (spatially averaged over individual spectra acquired at the four locations indicated in Fig. 2a) clearly showing well resolved molecular resonance peaks centered at −0.9 V, −0.5 V, +0.65 V and +1.1 V. A discernible region of low conductance is visible between the peaks located at −0.5 V and +0.65 V which can be attributed to the upper bound molecular states referred to as the highest occupied molecular orbital (HOMO) and lower bound molecular states referred to as the lowest unoccupied molecular orbital (LUMO), respectively. The emergence of sharper spectral features (Fig. 2b) for the regular C_60_ dimers adsorbed on the insulating spacer than on bare gold substantiates the preservation of intrinsic molecular states involved in electron transport. DFT based electronic structure calculations of the frontier molecular orbitals (denoted by blue arrows with their corresponding experimentally measured resonance peaks in Fig. 2b) reveal the HOMO to be localised over the fluorene molecular bridge linking the C_60_ anchor units and the LUMO to be localised on one of the C_60_ lobes. The localisation of the LUMO on the one C_60_ is indicative of weak electronic coupling between the C_60_ anchor units^25^. These results are consistent with previous DFT calculations on similar C_60_ dimer structures^24^,^25^. Based on DFT electronic structure calculations a HOMO-LUMO gap of 1.0 eV is computed which is in agreement with the experimentally measured value of (1.1 ± 0.1) eV obtained from measurements on ~30 regular C_60_ dimers.

## Quantifying Anomalies in Molecular Electronic Species

**Figure 3:**
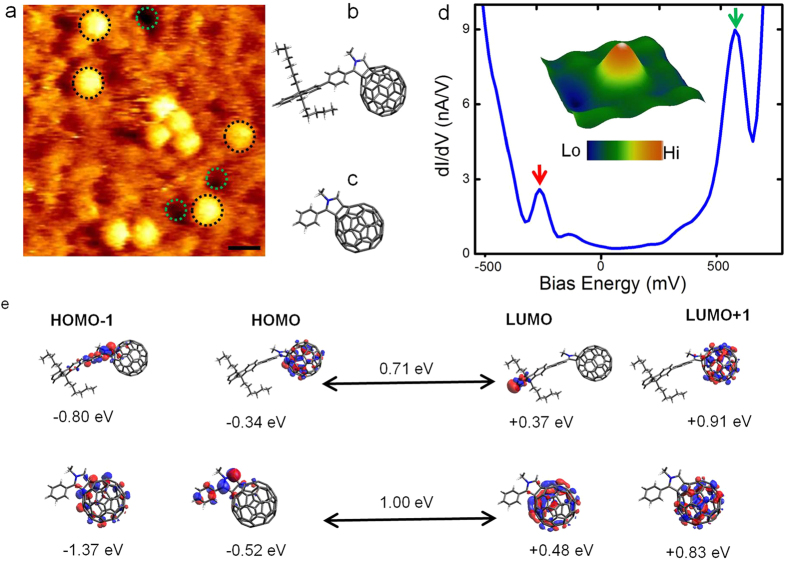
Spectroscopic analysis of structural variants. (**a**) Large-area *in situ* STM images showing the presence of individual C_60_ units (tunnelling parameters: I = 10 pA, V = 0.5 V, scale bar: 1 nm). The dashed green circles indicate the presence of naturally occurring pores on Au(111). (**b**,**c**) are molecular models for individual C_60_ molecules with long and short-chain lengths. (**d**) Spatially averaged dI/dV spectroscopic signature of individual C_60_ molecules (inset in panel d, is a high-resolution three-dimensional image of a single molecular unit over which spectroscopic data is acquired). (**e**) DFT based electronic structure calculations of the frontier molecular orbitals for representative neutral short-length and anionic long-length molecular segments attached to the fullerene with their respective conductance gaps values indicated above the black arrows separating the HOMO and LUMO eigen states. The full DFT data set is given in the Supplementary Section S8.

Interestingly, imaging on separate regions within the same sample revealed the presence of isolated, single C_60_ molecules. Figure 3a is an *in situ* STM image where such molecules are seen (circled in black). These individual molecules were observed to form clusters with neighboring molecules for a short-period of time, after which they disentangle. The bottom most molecular configuration in Fig. 3a resembles a dumbbell shape but is not a regular dimer molecule. It is instead two individual C_60_ molecules in close proximity, confirmed by their intercage separation of ~1 nm when the actual intercage separation for regular C_60_ dimers is ~2.5 nm. In addition, the individual C_60_ units observed in several *in situ* STM images structurally resemble pristine C_60_ molecules but were observed to have contrasting electronic signatures (Fig. 3d) when compared to actual pristine (non-functionalised) C_60_ molecules adsorbed on an alkyl-spacer (*n*-C_30_H_62_ in this case) coated Au(111) surface which we measured previously^36^ using the same experimental setup in liquids at room-temperature. The frontier molecular orbitals are well-resolved, thereby allowing the estimation of the energy gap between the frontier molecular states, HOMO (indicated by the red arrow) and the LUMO peaks (indicated by the green arrow).

This key experimental evidence on the functionality of individual molecules (dI/dV curve, Fig. 3d) suggests that the monomeric units detected in our study could still contain a segment of the molecular bridge that initially linked the two C_60_ anchors and are not actually pristine C_60_. Complementary standard chemical purity analytical tests also indicate that pristine, unfunctionalised C_60_ units are not present in the as-synthesized material (see section S1 Supplementary Section for electrochemical characterisation). For the current work, we consider two possible molecular configurations depicted in the molecular models of Fig. 3b,c. Note: The isolated monomers and the stable dimers pinned to the pores do not show any difference in respective spectral curves with their counterparts adsorbed on terrace edges or located on the terrace planes, indicating that the pores do not influence the measured electronic states of the molecules but are only topological peculiarities present on the surface.

As the entire STM imaging was performed using non-functionalised metal tips with no special chemical treatment to the tip-apex it has not been possible to determine the actual length of the molecular segment attached to the C_60_ cage similar to previous STM studies on functionalised C_60_ molecules^23^,^43^. Nonetheless, it should be feasible in future experiments to resolve this molecular segment linked to the monomers in real-space even in liquids using chemically terminated STM probes^44^ which is a well established technique to enhance sub-molecular resolution. The challenge we anticipate will be the reduction of molecular fluctuations at the metal-apex at room-temperature after the molecule has been transferred to the tip through lateral or vertical manipulation. The mechanical fluctuations the molecules undergoes at the metal tip apex would limit the lifetime of such molecular probes. However, this issue can be mitigated by fabricating and operating a single-molecule terminated STM tip in high-density liquids with C_60_ termination, based on previously discussed methodologies where translocation molecular motion is shown to be vastly reduced on solid surface using high-density liquids^36^.

## Density Functional Theory and STS Statistical Analysis

To gain deeper insights into the electronic structure of the molecules we performed DFT calculations and compared calculated and measured conductance gap values. Figure 3e shows the DFT computed HOMO-1, HOMO, LUMO and LUMO + 1 eigenstates for the individual C_60_ molecules with varying molecular chain length. A detailed description of the calculations and the computed electronic structures is provided in the Supporting Section S8. In general, the high-lying occupied orbitals are located on the molecular segment anchored to the C_60_ cage while low-lying unoccupied levels are localised over the C_60_ cage of the molecular complex, consistent with previous DFT reports on fullerene anchor based molecular complexes^24^,^25^,^45^,^46^. The calculated conductance gaps (based on the energy difference between the frontier molecular orbitals) for single molecules are (0.8 ± 0.3) eV are in good agreement with the structure-and time-averaged STS measured conductance gap values of (0.8 ± 0.2) eV. Random conformational changes and structural fluctuations cannot be totally excluded during STS measurements at room-temperature. However, owing to the high density of the liquid we have used, the translational motion of the molecule is reduced to a certain extent (if a very low boiling point solvent is used then solvent drying effects will cause additional fluctuations in the molecules). We did observe noisy spectral curves during our STS measurements, usually resulting from tip-contamination (sometimes arising from the organic spacer layer, seen from the disruption of the alkane spacer layer), and we have been careful to exclude these data from our analysis. We have used several tips prepared using identical protocols to acquire the STS data and do not find any trend for differences in spectral line shape as a function of the tip employed.

Based on experimental evidence and control measurements (see Supplementary Section S7), we suggest that the small population of single C_60_ hybrid molecules may stem from minute impurities arising during synthesis (although undetected in bulk purity tests S1 Supplementary Information, as the C_60_ lobes are still tethetered with a molecular segment). The possibility of the molecular backbone rupture during the landing of the molecules from liquid-phase onto solid surfaces, cannot be totally ignored. Such cracking of molecular backbone has been previously reported for large macromolecules and attributed to variations in the local interactions of the different segments of the molecule with the underlying surface^47^. Tip-induced molecular disintegration can be excluded as imaging was performed under low-biases (0.2–0.5 V) and low-tunnel current set points (2–25 pA) to exclude any tip related molecular fragmentation^48^, and the regular dimer molecules remained structurally stable even under high-bias conditions (+2 V). Earlier studies have described at length the role of the tunnelling electrons^49^ and tip-molecule interaction distance^50^ in inducing molecular motion and dissociation. Furthermore, when monitoring molecular structure and motion, we take extreme precautions (optimal imaging speed and low-tunnel current setpoints) to circumvent tip-induced molecular drag-drop, electrically driving the molecules along the surface or breaking of the regular fullerene dimers as a result of tip-interaction.

**Figure 4:**
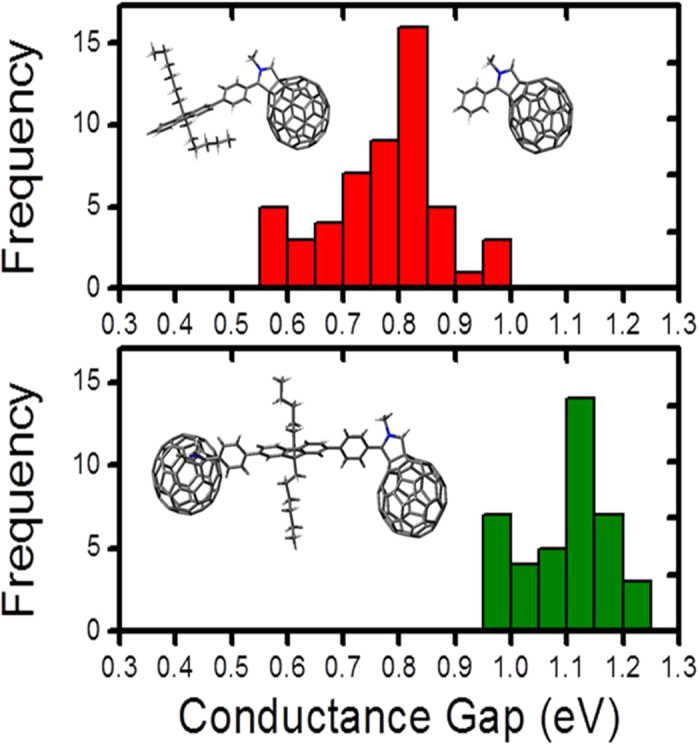
Statistical binning of conductance gap values. Statistical analysis of conductance gap distributions for individual monomers (red histogram) and regular dimers (green histogram) based on *in situ* STS measurements. The single molecules include both the long and short molecular segments as depicted in the molecular structures.

From the local point probe spectroscopic approach we observe a clear distinction between the electronic signatures for the regular dimers (Fig. 2b) and monomeric units (Fig. 3d). Analysing the peak positions of the frontier molecular orbitals for a large population of molecules for each case we map the spread in the conductance gaps that is the energy difference between the HOMO and LUMO derived molecular resonance peaks with respect to the Fermi edge. Figure 4 summarizes the results of statistical analysis of the conductance gap values individual molecules (red histogram, the binning process does not discriminate between long and short-broken dimers) and the distribution of the measured conductance values for the regular C_60_ dimers is also shown in the same panel (green histogram) for direct comparison between the different molecular species. We derive a mean conductance gap value (0.8 ± 0.2) eV for the individual molecules which is lower than the mean conductance gap of (1.1 ± 0.1) eV for the regular C_60_ dimers, and in agreement with the DFT computed HOMO-LUMO gaps.

## Summary

Although cooperative effects between molecules has been suggested as a possibility when interpreting line shapes in conductance histograms^16^, the trend we observe in the conductance spread for molecular complexes has not been previously reported. We find strong evidence for the existence of mixed electronic species in our experiments, and such a diversity within a standard set of molecules with varying chemical and electronic structures can be expected to have manifold contact geometries with the metal electrodes resulting in a larger spread in the measured conductance. We highlight the importance of high-precision *in situ* profiling of the electronic structure, molecular structural stability and intermolecular interaction events occurring in the presence of liquid at room-temperature. The impact of such mixed electronic species warrants consideration when interpreting the information content from single channel peaks in molecular conductance histograms^5^,^8^,^9^,^10^,^16^,^20^,^21^ and during analysis of random telegraphic switching of molecular conductance behavior^6^ registered using experimental techniques that lack real-space information.

## Additional Information

**How to cite this article**: Nirmalraj, P. *et al.* Fingerprinting Electronic Molecular Complexes in Liquid. *Sci. Rep.* **6**, 19009; doi: 10.1038/srep19009 (2016).

## Supplementary Material

Supplementary Information

## References

[b1] Makk P. *et al.* Correlation analysis of atomic and single-molecule junction conductance. ACS Nano 6, 3411–3423 (2012). 22397391 10.1021/nn300440f

[b2] Néel N., Kröger J. & Berndt R. Two-Level Conductance Fluctuations of a Single-Molecule Junction. Nano Lett 11, 3593–3596 (2011). 21854026 10.1021/nl201327c

[b3] Basch H., Cohen R. & Ratner M. A. Interface Geometry and Molecular Junction Conductance: Geometric Fluctuation and Stochastic Switching. Nano Lett 5, 1668–1675 (2005). 16159203 10.1021/nl050702s

[b4] Hu Y., Zhu Y., Gao H. & Guo H. Conductance of an Ensemble of Molecular Wires: A Statistical Analysis. Phys Rev Lett 95, 156803 (2005). 16241750 10.1103/PhysRevLett.95.156803

[b5] Venkataraman L. *et al.* Single-Molecule Circuits with Well-Defined Molecular Conductance. Nano Lett 6, 458–462 (2006). 16522042 10.1021/nl052373+

[b6] Tao N. J. Electron transport in molecular junctions. Nat Nano 1, 173–181 (2006). 10.1038/nnano.2006.130 18654182

[b7] Mishchenko A. *et al.* Influence of Conformation on Conductance of Biphenyl-Dithiol Single-Molecule Contacts. Nano Lett 10, 156–163 (2009). 20025266 10.1021/nl903084b

[b8] Venkataraman L., Klare J. E., Nuckolls C., Hybertsen M. S. & Steigerwald M. L. Dependence of single-molecule junction conductance on molecular conformation. Nature 442, 904–907 (2006). 16929295 10.1038/nature05037

[b9] Wassel R. A., Fuierer R. R., Kim N. & Gorman C. B. Stochastic Variation in Conductance on the Nanometer Scale: A General Phenomenon. Nano Lett 3, 1617–1620 (2003).

[b10] French W. R. *et al.* Structural Origins of Conductance Fluctuations in Gold–Thiolate Molecular Transport Junctions. J Phys Chem Lett 4, 887–891 (2013). 26291351 10.1021/jz4001104

[b11] Kuznetsov A. M., Sommer-Larsen P. & Ulstrup J. Resonance and environmental fluctuation effects in STM currents through large adsorbed molecules. Surf Sci 275, 52–64 (1992).

[b12] Selzer Y. *et al.* Effect of Local Environment on Molecular Conduction: Isolated Molecule versus Self-Assembled Monolayer. Nano Lett 5, 61–65 (2004). 15792413 10.1021/nl048372j

[b13] Long D. P. *et al.* Effects of hydration on molecular junction transport. Nat Mater 5, 901–908 (2006). 17041584 10.1038/nmat1754

[b14] He H. *et al.* A Conducting Polymer Nanojunction Switch. J Am Chem Soc 123, 7730–7731 (2001). 11481008 10.1021/ja016264i

[b15] Di Ventra M., Kim S. G., Pantelides S. T. & Lang N. D. Temperature effects on the transport properties of molecules. Phys Rev Lett 86, 288–291 (2001). 11177813 10.1103/PhysRevLett.86.288

[b16] Reuter M. G., Hersam M. C., Seideman T. & Ratner M. A. Signatures of Cooperative Effects and Transport Mechanisms in Conductance Histograms. Nano Lett 12, 2243–2248 (2012). 22494042 10.1021/nl204379j

[b17] Chen W. *et al.* Aromaticity Decreases Single-Molecule Junction Conductance. J Am Chem Soc 136, 918–920 (2014). 24397414 10.1021/ja411143s

[b18] Liu S. P. *et al.* Electronic transport through short dsDNA measured with mechanically controlled break junctions: New thiol–gold binding protocol improves conductance. phys status solidi (b) 250, 2342–2348 (2013).

[b19] Martin C. A. *et al.* Fullerene-Based Anchoring Groups for Molecular Electronics. J Am Chem Soc 130, 13198–13199 (2008). 18788808 10.1021/ja804699a

[b20] Lortscher E. *et al.* Bonding and electronic transport properties of fullerene and fullerene derivatives in break-junction geometries. Small 9, 209–214 (2013). 23008229 10.1002/smll.201201688

[b21] Fock J. *et al.* A statistical approach to inelastic electron tunneling spectroscopy on fullerene-terminated molecules. Phys Chem Chem Phys 13, 14325–14332 (2011). 21698320 10.1039/c1cp20861f

[b22] Gillemot K. *et al.* A Detailed Experimental and Theoretical Study into the Properties of C_60_ Dumbbell Junctions. Small 9, 3812–3822 (2013). 23630169 10.1002/smll.201300310

[b23] Leary E. *et al.* Unambiguous One-Molecule Conductance Measurements under Ambient Conditions. Nano Lett 11, 2236–2241 (2011). 21548597 10.1021/nl200294s

[b24] Bilan S., Zotti L. A., Pauly F. & Cuevas J. C. Theoretical study of the charge transport through C_60_ based single-molecule junctions. Phys Rev B 85, 205403 (2012).

[b25] Markussen T., Settnes M. & Thygesen K. S. Robust conductance of dumbbell molecular junctions with fullerene anchoring groups. J Chem Phys 135, 144104–144106 (2011). 22010695 10.1063/1.3646510

[b26] De Feyter S. & De Schryver F. C. Self-assembly at the liquid/solid interface: STM reveals. J Phys Chem B 109, 4290–4302 (2005). 16851494 10.1021/jp045298k

[b27] Uji I. H. *et al.* Scanning tunneling microscopy and spectroscopy of donor-acceptor-donor triads at the liquid/solid interface. Chem Phys Chem 6, 2389–2395 (2005). 16273572 10.1002/cphc.200500241

[b28] Elemans J. A. A. W. & De Feyter S. Structure and function revealed with submolecular resolution at the liquid-solid interface. Soft Matter 5, 721–735 (2009).

[b29] Marchenko O. & Cousty J. Molecule length-induced reentrant self-organization of alkanes in monolayers adsorbed on Au(111). Phys Rev Lett 84, 5363–5366 (2000). 10990944 10.1103/PhysRevLett.84.5363

[b30] Katsonis N., Marchenko A. & Fichou D. Dynamics and spectroscopy of single C_60_ molecules adsorbed on Au(111) at the liquid–solid interface. J Photochem Photobiol A Chem 158, 101–104 (2003).

[b31] Yang Y.-C., Taranovskyy A. & Magnussen O. M. Thiolate-Induced Metal Adatom Trapping at Solid–Liquid Interfaces. Angew Chem Int Ed Engl 51, 1966–1969 (2012). 22250016 10.1002/anie.201106584

[b32] Yang Y.-C. & Magnussen O. M. Quantitative studies of adsorbate dynamics at noble metal electrodes by *in situ* Video-STM. Phys Chem Chem Phys 15, 12480–12487 (2013). 23652411 10.1039/c3cp51027a

[b33] Nirmalraj P. N., Thompson D. & Riel H. E. Capturing the embryonic stages of self-assembly—design rules for molecular computation. Sci. Rep. 5, 10116 (2015). 25960364 10.1038/srep10116 PMC4650799

[b34] Kampschulte L. *et al.* Thermodynamical Equilibrium of Binary Supramolecular Networks at the Liquid−Solid Interface. J Am Chem Soc 130, 8502–8507 (2008). 18533654 10.1021/ja801883t

[b35] Nirmalraj P. N., Schmid H., Gotsmann B. & Riel H. Nanoscale Origin of Defects at Metal/Molecule Engineered Interfaces. Langmuir 29, 1340–1345 (2013). 23339343 10.1021/la3046109

[b36] Nirmalraj P. *et al.* Nanoelectrical analysis of single molecules and atomic-scale materials at the solid/liquid interface. Nat Mater 13, 947–953 (2014). 25129620 10.1038/nmat4060

[b37] Lortscher E., Widmer D. & Gotsmann B. Next-generation nanotechnology laboratories with simultaneous reduction of all relevant disturbances. Nanoscale 5, 10542–10549 (2013). 24056900 10.1039/c3nr03373b

[b38] Mishchenko A. *et al.* Single-Molecule Junctions Based on Nitrile-Terminated Biphenyls: A Promising New Anchoring Group. J Am Chem Soc 133, 184–187 (2010). 21155561 10.1021/ja107340t

[b39] Marchenko A., Lukyanets S. & Cousty J. Adsorption of alkanes on Au(111): Possible origin of STM contrast at the liquid/solid interface. Phys Rev B 65, 045414 (2002).

[b40] Hulsken B. & Speller S. Measuring the Au(111) surface state at the solid-liquid interface. Surf Sci 580, 95–100 (2005).

[b41] Bas H. *et al.* STM studies of the self-assembly of manganese porphyrin catalysts at the Au(111)− n -tetradecane interface. New J Phys 11, 083011 (2009).

[b42] Hugelmann M. Tunnel barrier height oscillations at the solid liquid interface. Surf Sci Lett 541, L643–638 (2003).

[b43] Diaconescu B. *et al.* Molecular Self-Assembly of Functionalised Fullerenes on a Metal Surface. Phys Rev Lett. 102, 056102 (2009). 19257526 10.1103/PhysRevLett.102.056102

[b44] Schull G., Frederiksen T., Brandbyge M. & Berndt R. Passing current through touching molecules. Phys Rev Lett 103, 206803 (2009). 20365999 10.1103/PhysRevLett.103.206803

[b45] Guldi D. M., Giacalone F., De la Torre G., Segura J. L. & Martín N. Topological Effects of a Rigid Chiral Spacer on the Electronic Interactions in Donor–Acceptor Ensembles. Eur J Chem A 11, 7199–7210 (2005). 10.1002/chem.200500209 16163762

[b46] Molina-Ontoria A. *et al.* Self-Association and Electron Transfer in Donor−Acceptor Dyads Connected by meta-Substituted Oligomers. J Am Soc 131, 12218–12229 (2009). 10.1021/ja9024269 19705914

[b47] Sheiko S. S. *et al.* Adsorption-induced scission of carbon-carbon bonds. Nature 440, 191–194 (2006). 16525468 10.1038/nature04576

[b48] Dujardin G., Walkup R. E. & Avouris P. Dissociation of individual molecules with electrons from the tip of a scanning tunneling microscope. Science 255, 1232–1235 (1992). 17816830 10.1126/science.255.5049.1232

[b49] Stipe B. C. *et al.* Single-Molecule Dissociation by Tunneling Electrons. Phys Rev Lett 78, 4410–4413 (1997).

[b50] Böhringer M., Schneider W.-D. & Berndt R. Scanning tunneling microscope-induced molecular motion and its effect on the image formation. Surf Sci 408, 72–85 (1998).

