# Supplementary material for: Fingerprinting Electronic Molecular Complexes in Liquid
Source: Sci Rep. 2016 Jan 8;6:19009. doi: 10.1038/srep19009 (PMC4705545; doi:10.1038/srep19009)
Supplement: Supplementary Information [file srep19009-s1.pdf]

Fingerprinting Electronic Molecular Complexes in Liquid

Peter Nirmalraj[1], Andrea La Rosa[2], Damien Thompson [3,4], Marilyne Sousa[1], Nazario Martin[2], Bernd Gotsmann[1] and Heike Riel[1]

[1] IBM Research – Zurich, Säumerstrasse 4, CH- 8803 Rüschlikon, Switzerland.

[2] Departmento de Quimica Organica, Facultad de Quimica, Universidad Complutense de Madrid, E-28040, Madrid, Spain.

[3] Department of Physics and Energy, University of Limerick, Ireland.

[4] Materials and Surface Science Institute, University of Limerick, Ireland.

S1. Chemical synthesis and electrochemical analysis of C_{60} dimers.

First Step.

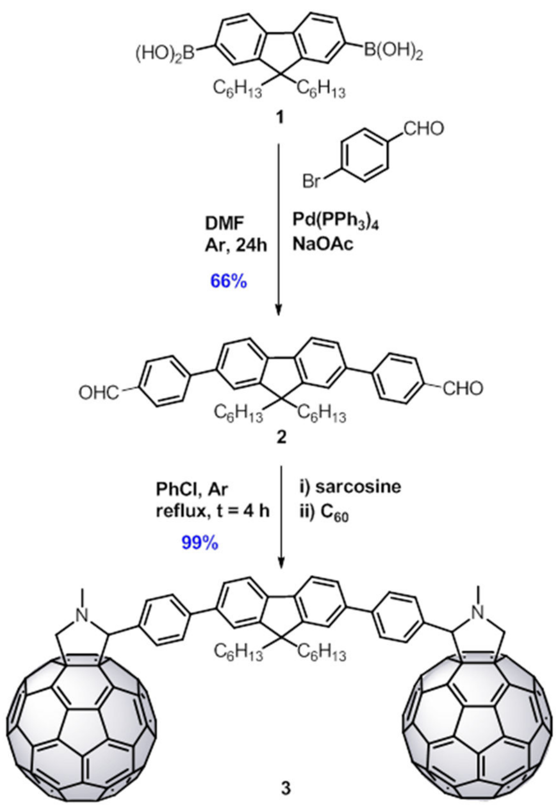

Scheme 1. Reaction scheme for the synthesis of compound **3**.

9,9’-dihexylfluorene-2,7-diboronic acid **1** (0.5 eqs, 1.0 g, 2.368 mmol), 4-bromo-benzaldehyde (1.0 eq, 877 mg, 4.737 mmol) and AcONa (20 eqs, 6.44 g, 47,4 mmol) were dissolved in 150 mL of dry DMF and purged with argon for 1h. After this period, $Pd(PPh_3)_4$ (10% mol, 0.274 g, 0.2369 mmol) was rapidly added to the solution and the solution was purged for a further 30 minutes in argon flow before heating it at 100 ºC for 15 h. The solution was cooled at room temperature and eluted with iced water. It was extracted with 3x100 mL of DCM and organic layers were collected, dried on $MgSO_4$, filtered and concentrated to dryness to obtain a crude material as dense oil. It was passed

by silica gel chromatography eluting with a gradient from pure Cy to AcOEt. 850 mg (1.566 mmol) of desired product was obtained as yellow dense oil in 66% yield.

Characterisation of compound **2**

The spectroscopic data of this compound are already collected from previous work [1]:

Second Step.

It has been performed using the general approach of Prato's reaction [2] to obtain the dumbbell-shaped molecules.

General Procedure.

In a round bottom flask, the corresponding aryl dialdehyde (1.0 eq) and sarcosine (8.0 eqs) are dissolved in 40 mL of chlorobenzene. C_{60} (8.0 eqs) is dissolved in further 40 mL of chlorobenzene and sonicated for 15 minutes. C_{60} solution is poured into the first one and heated at reflux temperature for 4 h. After cooling it to room temperature, it is washed with 2x150 mL of H_2O and then washed with 2x50 mL of brine. The organic layer is collected, dried over $MgSO_4$, filtered and concentrated to dryness. Crude material is purified by silica gel chromatography eluting with pure carbon disulphide (CS_2) to remove the unreacted C_{60}, followed by a mixture of CS_2:DCM with different composition according to the separation properties of the resulting pyrrolidine dumbbell. The reported reaction yield was quantitative and it is related to the consumed amount of the aryl dialdehyde (as limitating agent) and not to the recovery of the C_{60} unreacted.

Characterization of compound **3**

Mp = > 300 °C

^{1}H-NMR (*$CDCl_3$, 700 MHz, 25 °C*): δ = 8.16 (bs, **4H**), 7.96 (bd, **6H**), 7.81 (d, *J = 8.1 Hz*, **2H$_{\text{fluorene}}$**), 7.77 (s, **2H$_{\text{fluorene}}$**), 5.30 (d, *J = 9.4 Hz*, **2H**), 5.29 (s, **2H**), 4.61 (d, *J = 9.4 Hz*,

2H), 3.17 (s, **6H**), 2.31 (m, **4H**), 1.35 (m, **12H**), 1.05 (t, *J = 7.5 Hz*, **6H**), 0.96 (bs, **4H**) ppm.

^{13}C-NMR (*$CDCl_3$, 175 MHz, 25 °C*): δ = 156.5, 154.2, 153.7, 153.5, 151.9, 147.6, 147.1, 146.7, 146.7, 146.60, 146.5, 146.4, 146.2, 146.1, 146.0, 145.8, 145.7, 145.6, 145.5, 145.0, 144.7, 143.5, 143.4, 143.0, 142.9, 142.6, 142.5, 142.4, 142.3, 142.0, 141.9, 140.6, 140.0, 139.8, 126.5, 121.6, 120.7, 83.7, 70.5, 69.3, 55.4, 41.2, 40.4, 32.4, 30.7, 24.7, 23.7, 15.0 ppm. m/z (MALDI-ToF, negative): Calculated Mass for $C_{163}H_{52}N_2$: 2036.413 [M]; Found Mass: 2035.978 $[M-1]^-$

HPLC: Buckyprep type Waters 4.6x250 mm; 1.0 ml/min flow rate; toluene:i-Prop/9:1

The probe solution has been prepared dissolving few mgs of compound **3** in few mLs of carbon disulphide. Filtered and use as neat sample for the HPLC analysis.

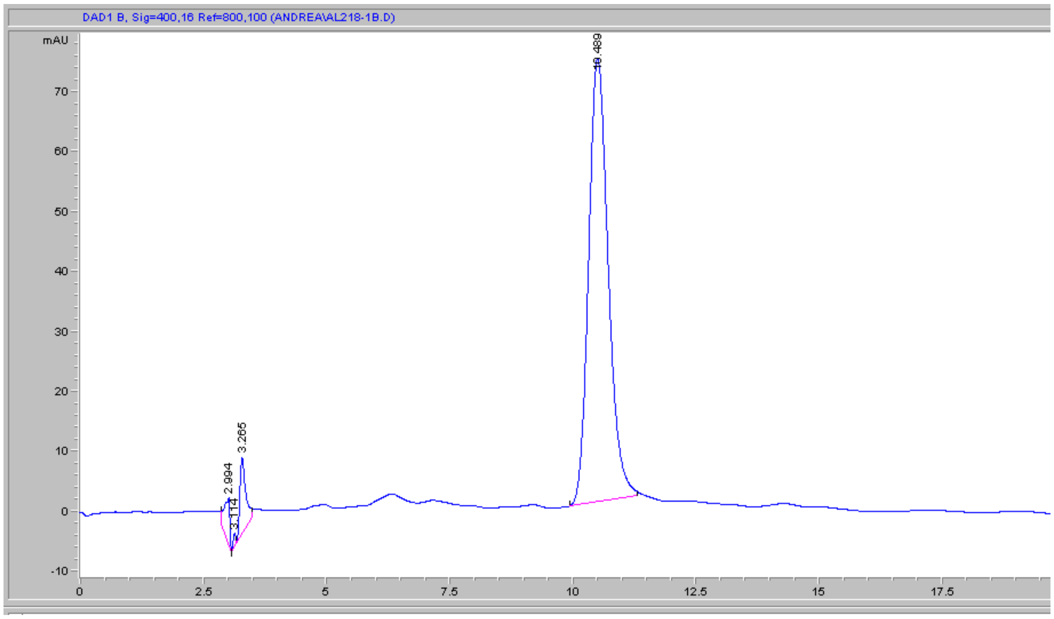

Figure 1. HPLC analysis of compound 3 and its stability.

At 10.489 min, the peak of the eluted compound has been envisaged without any further peaks referring to the decomposition of the material.

CV measurements

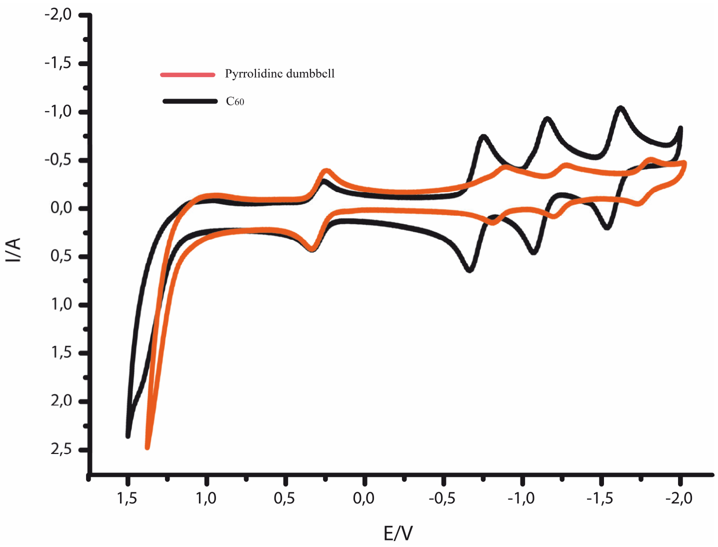

Figure 2. Cyclic voltammetry [V vs Fc/Fc^+] of C_{60} and pyrrolidine dumbbell **3**. Working electrode: GCE; reference electrode: Ag/Ag^+; counter electrode: Pt; supporting electrolyte: 0.1M Bu_4NClO_4; scan rate: 100 mVs^{-1}; solvent: *o*-DCB/MeCN (4 : 1 v/v).

Compounds	E^1_{red}	E^2_{red}	E^3_{red}	E^4_{red}	E^5_{red}	E^1_{ox}	E^2_{ox}
C_{60}	-1.00	-1.41	-1.87				
**3**	-1.13	-1.52	-2.05			—	—

Table 1. Reduction potential values for pristine C_{60} and compound **3**. The difference in the first reduction potential wave is indicative of the saturation of one double bond of C_{60} with consequent destabilisation of the LUMO. It is more difficult to reduce the dumbbell (compound **3**) under these conditions.

The π-π interaction between the dimer molecule and the stationary phase of the column allows us to distinguish the dimers from traces of pristine C_{60} units. The dimer molecules were found to be stable under ambient conditions and upon light exposure. Owing to the less number of double bonds in the C_{60} cage with respect to the dimer molecule, the

pristine fullerenes are less retained in the buckyprep column. This implies that the pristine fullerenes will be rejected out of the column with retention time smaller than that related to the dimer molecule. No additional peaks are visible in the HPLC chromatogram.

Dispersion and deposition of C_{60} dimers.

The C_{60} dimers in powder form were solublised in *n*-tetradecane (99% purity, high grade solvent, Sigma Aldrich) to a concentration of 0.5 mM. The solution was subjected to a 30 second gentle bath sonication prior to being injected onto the organic-spacer coated Au(111) placed in the liquid-cell. For valid comparative analysis the same dispersion was used for in situ STM/STS measurements of C_{60} dimers deposited on bare Au(111). Solublising the molecules in tetrahydrofuran (THF), toluene and dichlorobenzene without any bath sonication and drop casting them on metals and spacer-coated metals resulted in similar results (presence of functionalised-single fullerenes in addition to the regular dimers) from molecules prepared in separate batches (to exclude any particular synthesis differences). This information clearly indicates that irrespective of the solvent used or the synthesis methodology adopted in dispersing these molecular complexes the presence of functionalised-single fullerenes was seen invariably in all batches.

S2. Fabrication and characterisation of the organic spacer layer.

The *n*-$C_{14}H_{30}$ spacer layer was formed on freshly prepared Au(111) (sample size: 1cm × 1cm) by spray coating (Dual action, Evolution Airbrush, Harder Steenbeck, nozzle size, 0.15 mm) with 300 μL of *n*-tetradecane solvent to form an ultra-thin film of *n*-$C_{14}H_{30}$ molecules. The distance between the gold substrate and the nozzle of the spray gun during the deposition was ~100 mm under a compressed nitrogen flow rate of ~1.5 mbar. The best results were obtained when the substrate was held at room-temperature and a single spray-pass was performed. The organic layer coated Au (111) sample was kept in

the fume cupboard for 30 minutes before deposition of the C_{60} dimer molecules. On top of the *in situ* STM characterisation of the organic spacer layer to probe structural arrangements of the molecules within the lamellae, ex-situ high-resolution ellipsometry was performed for large-area characterisation of the spacer layer. Measurements were carried out with a variable angle spectroscopic ellipsometry VASE® from J.A. Woollam Co. equipped with focusing probes in order to reduce the beam size to 200 micrometers. Au (111) and *n*-$C_{14}H_{30}$/ Au (111) were measured ex-situ between 300 nm and 1600 nm at 65 and 70 degrees C. To determine the thickness of self assembled monolayers, the best way to proceed, as described previously [3] is to simulate the data file corresponding to the difference between the raw data of the stack [n-$C_{14}H_{30}$/Au(111)] and the Au(111) substrate itself. An average file was then calculated for each sample and used for the simulations. To get rid of the typical correlation observed between optical indices and thickness on such ultra-thin films, the optical index of the *n*-$C_{14}H_{30}$ layer was fixed to its real component 'n' and the raw data were simulated for various thickness until a match was found between the simulation and the averaged raw file. $C_{14}H_{30}$ films were simulated using a Cauchy layer:

$$n = A_n + \frac{B_n}{\lambda^2} + \frac{C_n}{\lambda^4}$$

with An= 1.428 (value taken from www.Chemspider.com).The four averaged files were analysed together. In ultra-thin layers thickness and optical indices are correlated, and so one fixes the optical index of the *n*-$C_{14}H_{30}$ layer and simulates the data for various thickness until finding a match with the plots (Ψ_{C14H30}-Ψ_{Au}) and ((Δ_{C14H30}-Δ_{Au}). For this film, the optical index N was fixed to its real component An which is equal to 1.428

(value taken from www.Chemspider.com). Our successive adjustments led to a monolayer thickness of ~4 Angstroms for the *n*-$C_{14}H_{30}$ monolayer.

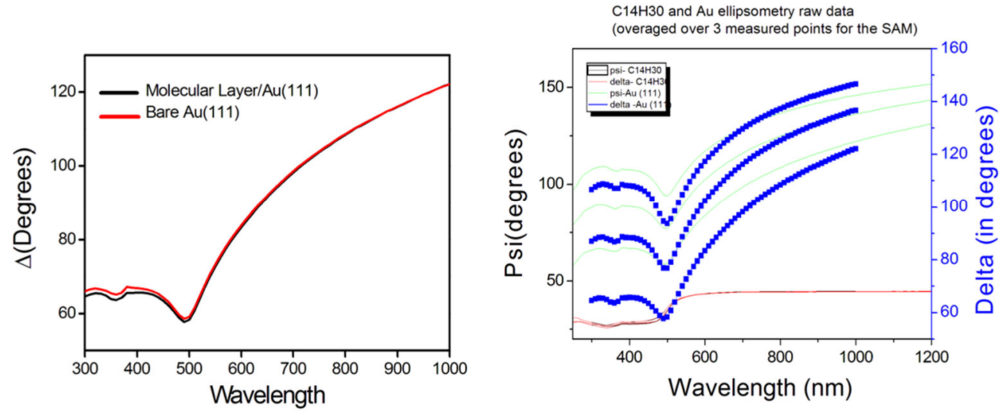

Figure. 3: Ellipsometry analysis of *n*-$C_{14}H_{30}$ molecular layer on Au(111).

S3. Physical properties of the liquid medium (*n*-tetradecane).

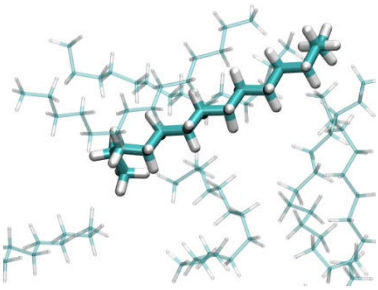

Figure 4: Computed intermolecular packing arrangements in bulk liquid *n*-tetradecane (employed for *in situ* STM/STS measurements). One molecule is displayed as thick sticks and surrounding molecules making sub-3 Å contacts in molecular dynamics simulations (described in section S9 below) are shown as semi-transparent thin sticks. More distant molecules and periodic image molecules are removed for clarity. Image generation and Tcl script-based trajectory analysis was performed using the VMD program [4].

Liquid Medium	Molecule Packing Energy (kcal/mol) *	Root Mean Square Fluctuations (Å) *	Dielectric Constant	Density (g/mL at 25 ° C	Boiling Point (° C)
n-tetradecane	-36.2 ± 0.9	2.5 ± 0.4	2.0	0.762	252

Table 2: Physical properties of the bulk *n*-tetradecane liquid.* Calculated from room temperature MD simulations of 100 molecules in a cubic box with periodic boundary conditions sampled at 1 atm pressure for 40 ns. Packing energy is the computed time-averaged van der Waals interaction energy between molecules; RMSF values are computed fluctuations in the carbon atom positions in the bulk liquid, measured over 50 equally-spaced structures taken during the final 10 ns of dynamics.

S4. *In situ* STM image of C_{60} dimer distribution.

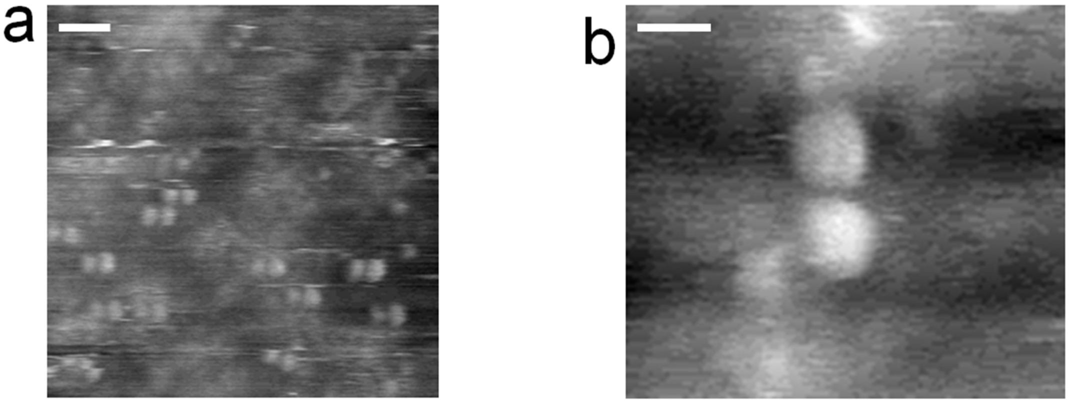

Figure 5: a, Large-area *in situ* STM image recorded immediately after drop-casting the dimer solution onto a Au(111) surface. Although traces of monomers are visible from the STM image, most of the structures represent a regular C_{60} dimer. (tunnelling parameters : I = 25 pA, V = 0.2 V scale bar: 5 nm). Such images were first recorded to provide an overview of the sample surface and to evaluate the distribution of C_{60} dimer versus individual molecule population. For the actual center-center distance calculations for the

regular dimers such images as shown in panel a were not used, rather a specific regular dimer is chosen from a large-area scan and zoomed-in as shown in panel b, which is imaged over time (tip-drift rate is ~1nm/min) and upon verifying it structural consistency, it is labeled as a regular dimer and the intercage distance is calculated. (tunnelling parameters : I = 10 pA, V = 0.2 V scale bar: 2 nm). The two *in situ* STM images shown above are raw data reported without any low-pass filtering or removal of random streaks during imaging in liquids. To improve the stability and minimize thermal drifts and reduce scanner hysteresis we continuously image over a reference sample, usually HOPG or Au(111) for a time period ranging from 5-6 hours before commencing measurements on the molecules deposited on Au(111) surface. Note: The ordering of the organic spacer layer previously apparent in dry conditions (Fig. 1c) is not visible in the large-area *in situ* STM images (Fig 2a-e). There could be two likely causes: 1) the formation of a disordered ad-layer of n-$C_{14}H_{30}$ molecules from the bulk solvent that prevents direct imaging of the ordered underlying spacer layer and 2) the limitations in resolving a multicomponent organic architecture with diverse electronic properties (conductive organic molecules located on a near-insulating organic spacer layer) using a common STM imaging parameter (bias energy and tunnel current set-point).

Methods

100 nm of Au (99.99 %, FHR Anlagenbau GmbH) was sputter deposited (Cluster system CS320S, Von Ardenne, Dresden) in an argon atmosphere onto freshly cleaved mica (1cm × 1cm) annealed at 450 $^{\circ}$C for four hours (High quality Grade V1, muscovite mica, purchased from SPI Supplies) at a rate of 10 Å s^{-1} and a base pressure of 2×10^{-7} mbar. Imaging and spectroscopic measurements were performed using a Nanoscope III a digital

instruments using an E- Scanner. For the STM/STS probes, mechanically cut Au wire (0.25 mm, Good Fellow GmbH) was used. The *in situ* STM imaging was performed under constant-current mode. The quality of the tip was constantly monitored by measuring dI/dV over bare gold substrates. No tip insulation is required as all the measurements in the current study were performed in non-conductive liquids, in cases requiring measurements in polar liquids, such as buffer solutions which is the native environment of biomolecules, additional insulation of the tip with Apiezon wax or high-density polyethylene is required and is also commercially available (N9803A, Keysight Technologies). A specialised liquid cell sample holder made of Teflon was used for the *in situ* measurements, the liquid-cell was thoroughly cleaned with acetone (30 minute bath sonication) and further rinsed in isopropyl alcohol prior to usage. All *in situ* STM/STS measurements were performed in n-$C_{14}H_{30}$ solvent medium (see Supplementary Section S3 for n-tetradecane properties). Full details of simulation models and methodologies are given in Supplementary Sections S7 and S8.

S5. Spatially averaged spectral signature for regular C_{60} dimer.

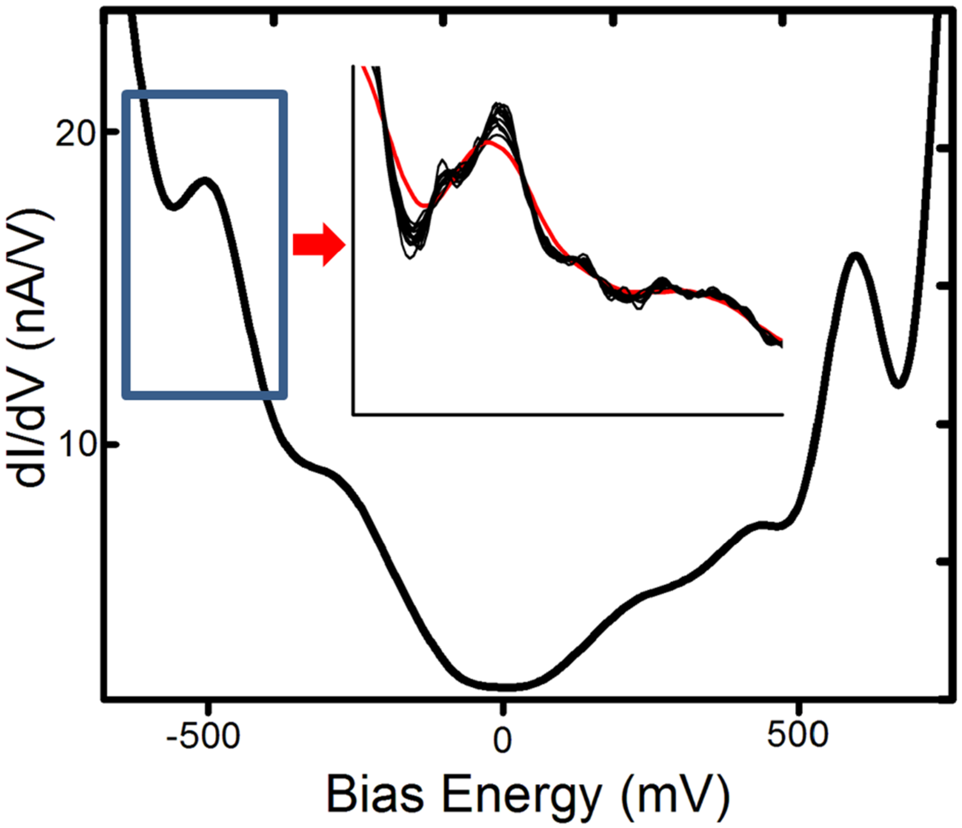

Figure 6: *In situ* STS spectral analysis of C_{60} dimers on spacer-coated Au(111). The spatially averaged dI/dV signal recorded on over 30 regular C_{60} dimers is shown in the above plot. The inset shows individual traces (black lines) of the HOMO peak (marked by blue box in the averaged spectrum) and the red trace shows the averaged curve over several individual traces.

S6: Details on spectroscopic measurement protocol over single molecules in liquids.

The molecule of interest (whether regular dimers or monomers) is first imaged with high-spatial resolution and its structural stability is verified through time-elapsed imaging before confirming the nature of the molecular species. Upon confirming the structure of the molecule to be studied, the feedback loop is shut off and the current (I) is measured as the voltage (V) is ramped between ±1 in steps of 100 mV. The acquisition time to record a single IV curve varies between 2-3 seconds. Although faster data acquisition time in possible with our STS setup, it generally results in noisier spectroscopic curves, hence the time frame of 2-3 seconds is maintained. After every 30 seconds of recording spectral data, the feedback loop is re-initiated and the molecular structure and tip-location is verified and on confirming that the molecule under study is stable and has not disintegrated during spectroscopic probing, the feedback loop is shut-off again and spectroscopic measurements is resumed. The spectral curves reported in this work (individual curves and spatially averaged curves) are selected only from reproducible (forward and reverse) data sets that comply with the set tunneling conditions. Our calibrated tip-drift rate is ~1 nm/min, this provides us a window to record the spectral curves within a timeframe before the tip drifts a distance equaling the radius of the C_{60} cage and in the case of the regular dimer molecules, the preamplifier is allowed to stabilize in-between recording the spectroscopic curves on different locations along the molecular length. The dI/dV spectral curves were obtained by numerical differentiation of the experimentally measured IV curves (point-to-point sliding averages). As all the spectroscopic measurements were performed on organic molecules, the issue of tip-contamination on the quality of the recorded spectral curves needs to be carefully analyzed. Hence we constantly monitor the quality of tips (check for double-tips) by imaging over a separate reference Au(111) sample. Also it is common practice that we constantly check the quality of the tip before and after using it to image adsorbed molecules. All reported curves were obtained with different metal tips and found to be reproducible. The variation in the measured spectral over separate molecules is minimal, although the line shape is retained, we observe minor shifts in the frontier molecular orbital peak positions reflected in the calculated experimental error gaps in each case.

The entire experimental setup is placed in well-controlled laboratory conditions shielded from extrinsic noise and mechanical vibrations, thereby providing additional stability during sensitive imaging and spectroscopic measurements [5].

S7. Control Measurements

We performed additional control experiments to investigate other possible factors that could stimulate the rupture of the molecular bridge. Based on our tests, the presence of the underlying organic spacer layer, bath sonication employed for solution synthesis, solvent purity and type can all be excluded as plausible reasons for the rupture of the molecular bridge. Monomers were also observed in control tests where the solution was prepared using high-purity solvents other than *n*-tetradecane (dimers solublised in tetrahydrofuran, toulene, decane and 1,2-dicholorobenzene were also tested). Deposition (under identical conditions) on bare gold without any short-bath sonication procedure also resulted in monomers with chemical functionalisation. However, the possible presence of a minute population of the functionalised monomer units in powder form, undetected by bulk chemical purity tests cannot be excluded.

S8. Density functional theory calculations.

Electronic structures were calculated using Gaussian09 (Gaussian 09, Revision D.01, Frisch, M. J. et al. Gaussian, Inc., Wallingford CT, 2009.) with the hybrid HF-DFT B3LYP functional [6] [7] and the 6-31G* basis set. Control simulations using a larger basis set, 6-311G**, gave the same HOMO-LUMO gap values to within 0.05 eV, as expected for these purely organic, low-polarity molecules. Good agreement between experiment and theory is found for the HOMO-LUMO separations (conductance gap values) for the dimer (1.1 ± 0.1 eV by STS, 1.0 eV by DFT) and monomer species (0.8 ± 0.2 eV by STS, 0.8 ± 0.2 eV by DFT). As described in the main text and in section S7 above it is difficult to pinpoint the origin of the monomers. Our DFT calculations give a computed biphenyl C-C bond dissociation energy in the dimer of +4.9 eV in agreement with estimates of 4.9 eV from experimental thermodynamic cycles (Jones, W. D. Mechanistic studies of transition metal-mediated C–C bond activation. Top. Curr. Chem. 346, 1–31 (2013)). However, formation of radical anion species (Fig. 8) and H-capped species (Fig. 9) reduces the DFT-computed energy penalty to 2.5-3.4 eV. On the other hand, radical cation monomers have much higher (unfavourable) formation energies. The calculated

electronic energies predict stabilisation of the short and long tailed monomers by -1.5 and -2.5 eV by formation of radical anion species but large penalties of +6.5 and +5.2 eV for formation of the corresponding radical cation short and long molecules. Thus we exclude cations from our analysis. We note that very recent work (see Wu et al. 2015 Nature 517, 449 and references therein) has shown gold-catalysed rupture of similar-strength biphenyl bonds in ambient conditions. In this work a strained C-C bond in biphenylene was ruptured with bond dissociation energy of 2.8 eV, similar to the net DFT energy cost of 2.5-3.4 eV calculated in the present work for formation of radical anion and H-terminated monomers from the regular dimer.

Figures 7-9 below show computed frontier and near-frontier molecular orbital surfaces for the regular dimer, the neutral monomers and radical anion monomers.

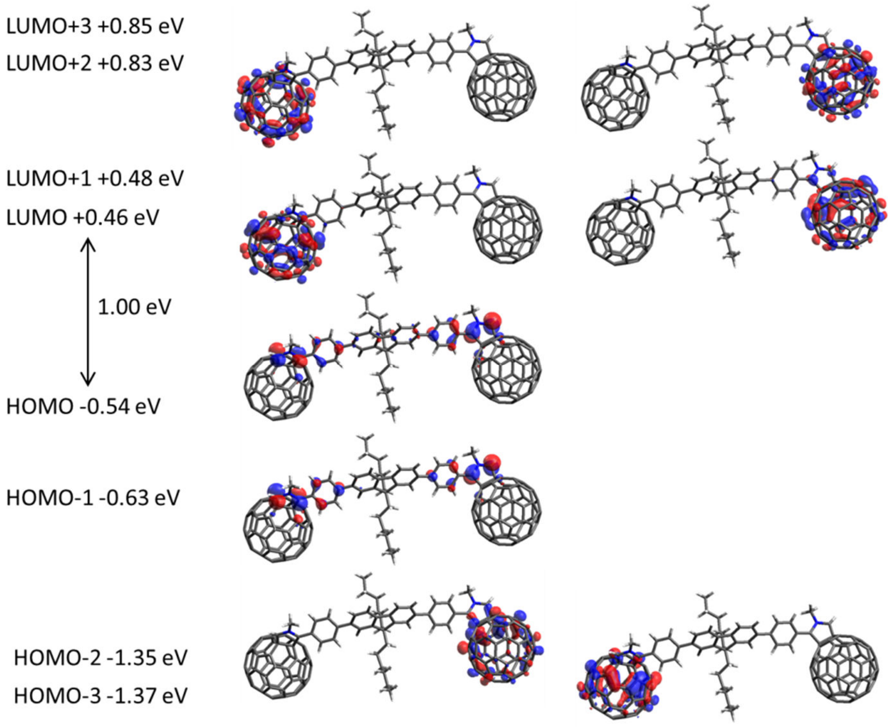

Figure 7: Calculated HOMO-3, HOMO-2, HOMO-1, HOMO, LUMO, LUMO+1, LUMO+2 and LUMO+3 surfaces for the regular dimer. Each wavefunction surface is visualised using the Avogadro code v1.1.[8] to view the Gaussian09 formatted checkpoint files using an isovalue of 0.03 atomic units, which highlights sites contributing strongly to the eigenstate. For ease of comparison with the measured experimental energy levels,

the computed eigenvalues are shifted upwards in energy by 3.81 eV, to set the HOMO eigenvalue at -0.5 eV, similar to the energy level of the first peak below the Fermi level in the experimental STS data. Given the closeness in energy of LUMO and LUMO+1 in the computed manifold, we assign the effective LUMO as a doubly degenerate state and interpret the eigenstate at +0.83 eV as the effective LUMO+1, *i.e.*, the second peak above the Fermi level in the STS spectrum.

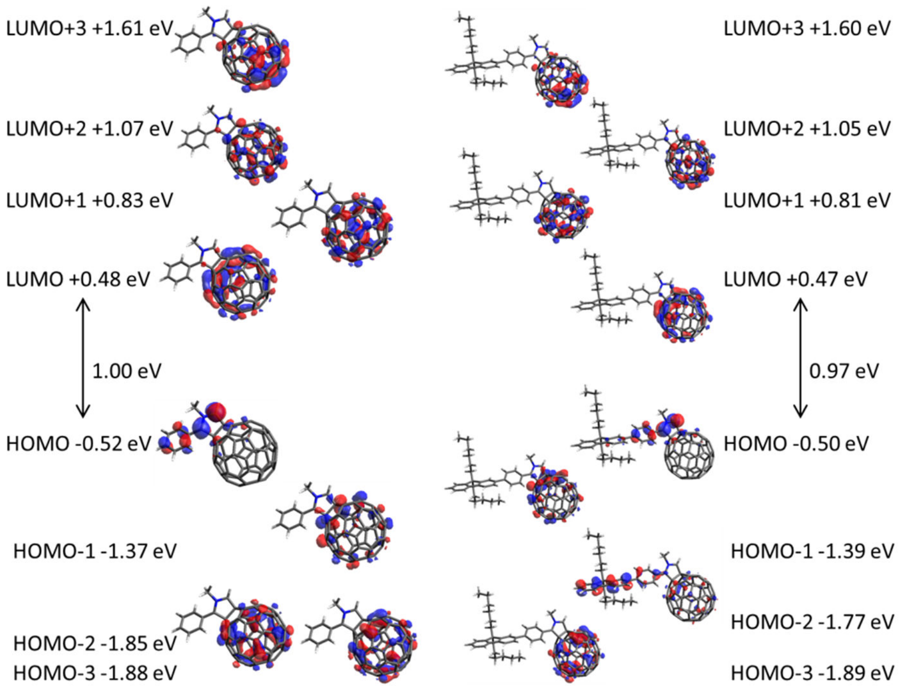

Figure 8: Calculated HOMO-3, HOMO-2, HOMO-1, HOMO, LUMO, LUMO+1, LUMO+2 and LUMO+3 surfaces for the neutral short and long tailed monomers.

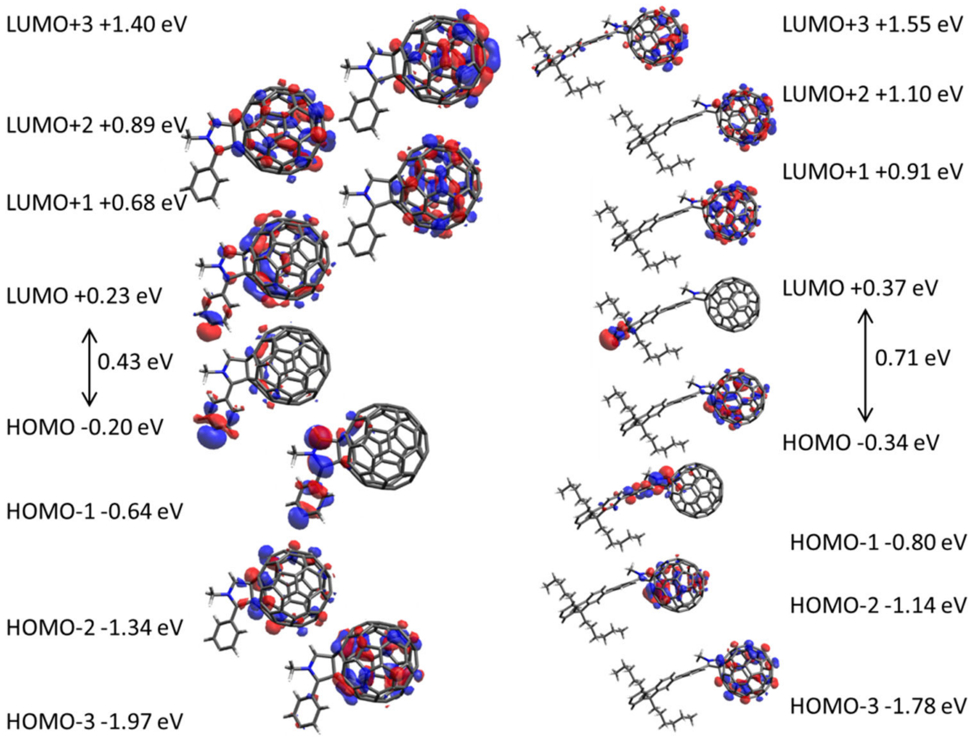

Figure 9: Calculated HOMO-3, HOMO-2, HOMO-1, HOMO, LUMO, LUMO+1, LUMO+2 and LUMO+3 surfaces for the anionic short and long broken fragments. For ease of comparison with the measured experimental energy levels, the computed eigenvalues are shifted upwards in energy by 1.11 eV.

S9 . Molecular dynamics simulations.

Fully atomistic molecular dynamics simulations were performed using the CHARMM force field [9] with an improved treatment of the Au van der Waals potential [10] and the NAMD code [11] to model formation of the tetradecane $C_{14}H_{30}$ spacer layer on Au(111) and subsequent adsorption of regular and individual dimer molecules on the spacer layer, in the presence of a top layer of bulk $C_{14}H_{30}$ solvent. The same force field and MD code was used to calculate the physicochemical properties of bulk $C_{14}H_{30}$ as described in section S3 above. The Au(111) surface contains a range of experimentally-observed pores. The pores in the model are 1 nm in diameter and have depths of 2.0, 3.3, 4.7 and 5.8 nm. Simulations were performed at room temperature using a two femtosecond

timestep and structures were sampled for 20 nanoseconds of molecular dynamics, following minimisation and one nanosecond of thermalisation and equilibration. A constant-density layer is formed within eight nanoseconds of room temperature molecular dynamics, from a starting structure of twenty 25-35-molecule clusters of n-$C_{14}H_{30}$ molecules suspended 0.5-1.0 nm above the Au(111) surface. Gold atoms were constrained to their starting crystallographic positions and kept neutral in the simulations. All interaction energies and rms deviation/fluctuation values reported were averaged over 100 equally-spaced snapshots sampled during the final 5 ns of 20 ns of equilibrated room temperature dynamics. Errors are given as standard deviations in the time- and molecule-averaged values.

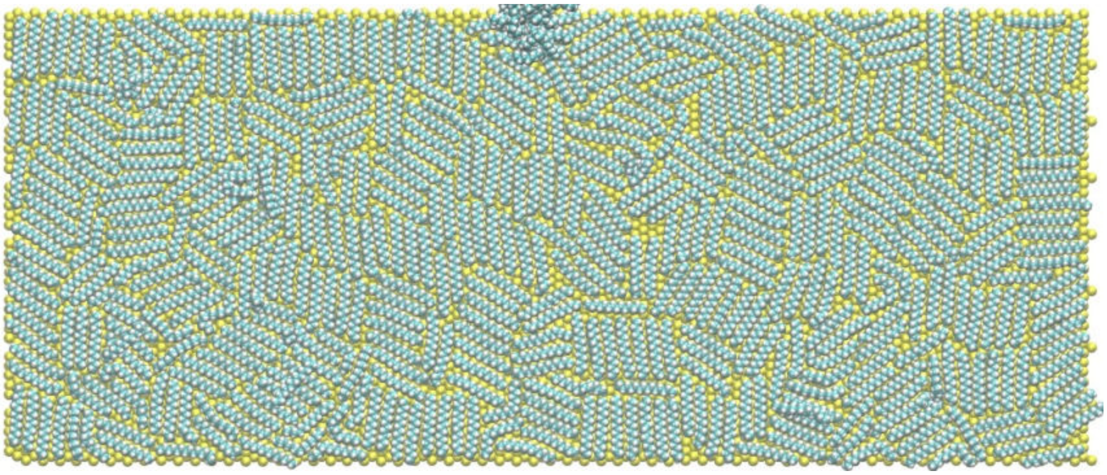

Figure 10: Top view of n-$C_{14}H_{30}$ monolayer on gold. Structure shown is the final structure formed following 20 ns of room temperature dynamics. All atoms shown as space-filling spheres with blue carbon atoms, white hydrogen atoms and yellow gold atoms.

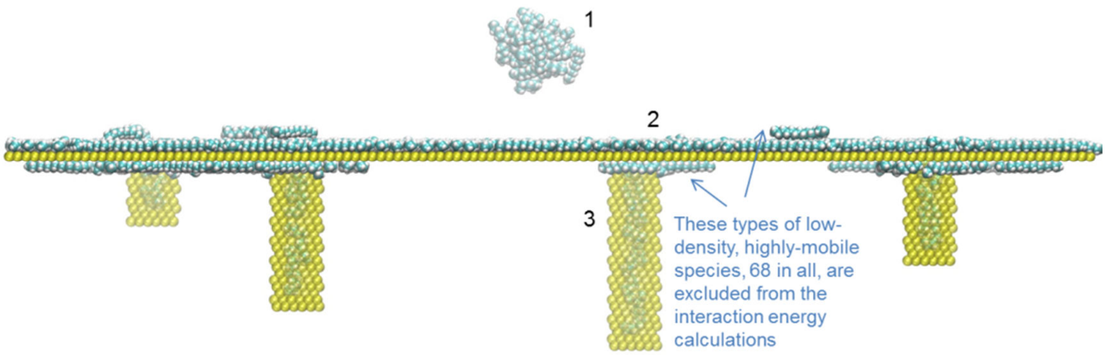

Figure 11: Side view of the self-assembled *n*-$C_{14}H_{30}$ spacer layer on gold. Regions labelled **1**, **2** and **3** show non-adsorbed clusters, spacer (mono) layer molecules adsorbed on Au(111) and in-pore assemblies of *n*-$C_{14}H_{30}$ molecules. Low-density regions on top of the spacer layer and on the underside of the gold are not included in the interaction energy calculations.

Table 3: Computed interaction energies for the spacer layer on gold (eV per *n*-$C_{14}H_{30}$ molecule)

Region	Molecule-molecule packing energy	Molecule-gold adsorption energy
1	-1.0 ± 0.1	N/A
2	-0.7 ± 0.1	-2.3 ± 0.3
3	-0.1 ± 0.0	-4.6 ± 0.4

Values shown are van der Waals interaction energies, averaged over 100 equally-spaced snapshots sampled during the final 5 ns of 20 ns of equilibrated room temperature dynamics. Errors are given as standard deviations in the time- and molecule-averaged energies. Molecule-averages are taken over the 500, 13 and 19 molecules respectively in the film, pores and suspended cluster in the 600-molecule simulation cell; note the 68 molecules that adsorb in low-density, highly-mobile configurations on the underside of Au(111) and on top of the alkane monolayer are shown in the structure but excluded from the energy calculations.

The alkane molecules fill the naturally-occurring pores in the gold substrate, with a strong net driving force for filling due to doubled molecule-gold adsorption energies in tubular rather than planar assemblies. Calculated physisorption energies are given in Table 3. Fig. 12 shows a representative structure of the regular, intact dimers adsorbed on the surface. Dumbbells do not diffuse significantly from their initial adsorption sites on

the surface, with calculated root mean square deviations (RMSD) of 0.7-2.4 Å from their landing sites over the course of 20 ns of molecular dynamics, indicating free molecular rotation, reorientation and optimisation of molecule-surface contacts without significant net center of mass molecular motion.

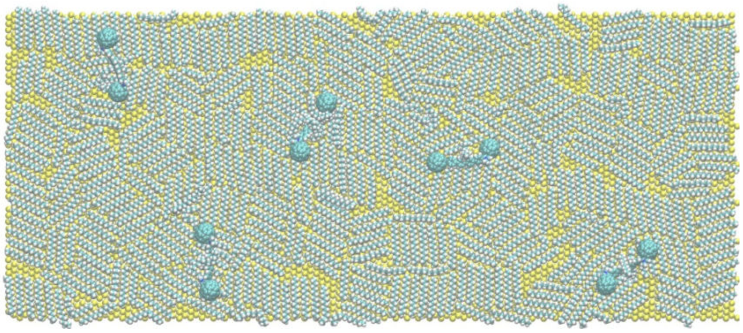

Figure 12: Top view of regular dimer molecules adsorbed on spacer-coated gold. Bulk n-$C_{14}H_{30}$ solvent molecules are omitted for clarity. The assembly shown is the final structure formed following 20 ns of room temperature dynamics on the pre-formed n-$C_{14}H_{30}$ monolayer on gold.

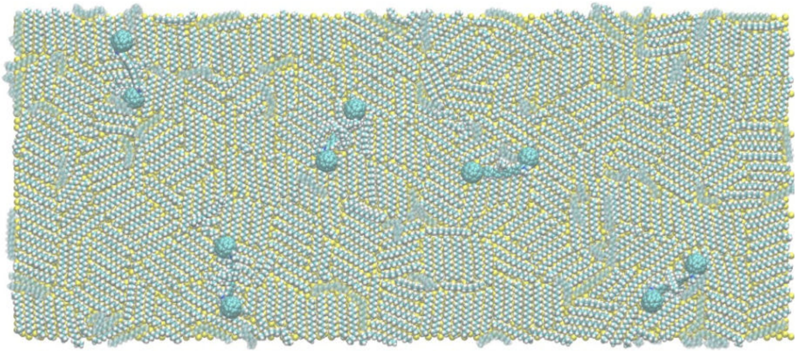

Figure 13: Top view of regular dimers adsorbed on the n-$C_{14}H_{30}$ monolayer on gold. Bulk n-$C_{14}H_{30}$ solvent molecules within 3 Å of gold are shown as transparent spheres, illustrating both the resistance of the pre-formed monolayer to extensive molecular exchange with bulk solvent molecules and the filling of the few atom-scale defects in the monolayer by solvent molecules.

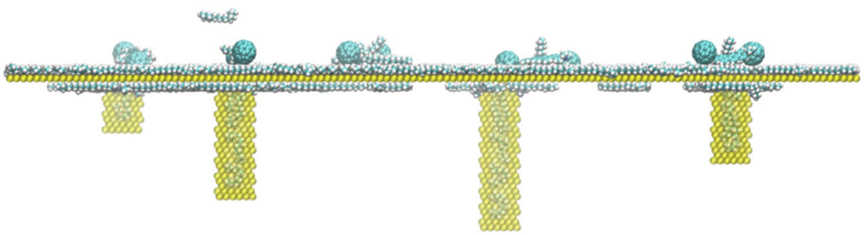

Figure 14: Side view of regular dimers adsorbed on the *n*-$C_{14}H_{30}$ monolayer on gold. Bulk *n*-$C_{14}H_{30}$ solvent molecules are omitted for clarity. Dimer molecules are numbered 1-5 reading left to right in the interaction energy calculations (Table 4).

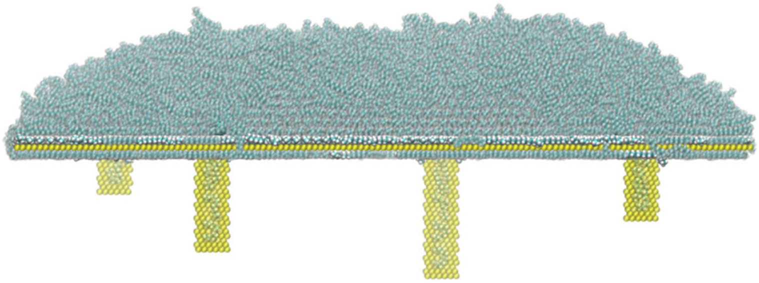

Figure 15: Side view of regular dimers adsorbed on the $C_{14}H_{30}$ monolayer on gold in the full 200,000-atom simulation cell. Bulk $C_{14}H_{30}$ solvent molecules are shown as transparent spheres.

Table 4: Computed interaction energies for regular dimer adsorption on spacer-coated gold (eV per dimer molecule)

Interface	Molecule No.				
	1	2	3	4	5
Dimer / gold	-2.1 ± 0.0	-0.4 ± 0.0	-0.8 ± 0.0	-4.4 ± 0.1	-0.8 ± 0.1
Dimer / spacer layer	-2.1 ± 0.0	-1.9 ± 0.1	-3.2 ± 0.1	-2.2 ± 0.1	-2.0 ± 0.1
Dimer / bulk solvent	-5.3 ± 0.2	-5.7 ± 0.2	-4.3 ± 0.1	-4.6 ± 0.1	-5.6 ± 0.3
Net dimer stabilisation less SAM disruption penalty of +1.0 ± 0.1 eV/mol per direct C_{60}-Au contact					
	-8.5 ± 0.3	-8.0 ± 0.3	-8.3 ± 0.2	-9.2 ± 0.3	-8.7 ± 0.4
	[one C_{60}-gold contact]			[two C_{60}-gold contacts]	

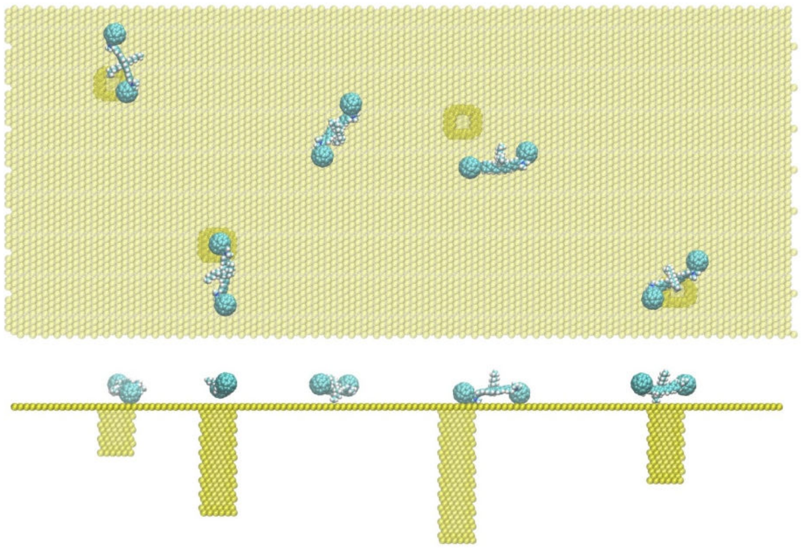

Figure 16: Adsorption modes of regular dimer with *n*-$C_{14}H_{30}$ molecules removed for clarity, showing the mix of on-spacer, on-gold and mixed adsorption modes present on the surface. Shown are top and side views of dumbbells adsorbed on the surface. Porous regions of gold are highlighted. Molecules are numbered 1-5 reading from left to right.

The calculated interaction energies indicate a small preference on average for the binding mode in which both fullerene units adsorb directly on gold. However, the error bars in the data show why all these binding modes are accessible in the simulations and indicate that on-SAM, on-gold and mixed binding modes are present at room temperature in the *n*-$C_{14}H_{30}$ cell. As shown previously for C_{60} molecules functionalised with exTTF-p-cyclophane (Nirmalraj, P. et al. Nanoelectrical analysis of single molecules and atomic-scale materials at the solid/liquid interface. Nat Mater 13, 947-953 (2014).), molecules are pinned to the surface. For the exTTF-p-cyclophane C_{60}, computed root mean square fluctuation (RMSF) values were (a) 2.4 ± 1.0 Å, (b) 1.8 ± 0.7 Å, and (c) 0.9 ± 0.6 Å respectively for functionalised C_{60} molecules adsorbed (a) on a *n*-$C_{30}H_{62}$ SAM in vacuum, (b) on the SAM in bulk silicone oil solvent and (c) adsorbed directly on gold. In the present study, computed RMSF values are further reduced due to the larger surface area of the molecule. For dimer molecules adsorbed on the *n*-$C_{14}H_{30}$ SAM in the presence

of bulk $C_{14}H_{30}$ with one or two direct C_{60}-gold contacts the RMSF values are 0.5 ± 0.3 and 0.4 ± 0.2 Å, and the RMSF values are 1.1 ± 0.5 Å for molecules adsorbed on the *n*-$C_{14}H_{30}$ SAM without any direct molecule-gold contacts. Hence the divalent molecules studied here that are adsorbed in and on the $C_{14}H_{30}$ spacer layer in bulk $C_{14}H_{30}$ are pinned to the *n*-$C_{14}H_{30}$ SAM as strongly as the mono-functionalised C_{60} was pinned directly to gold in bulk silicone oil in the earlier study. We modelled also the adsorption of the other species present in the experiments, functionalised monomers, as described below. We model the surface adsorption and dynamics of monomers in neutral and radical anion states (see DFT calculations in section S8 above).

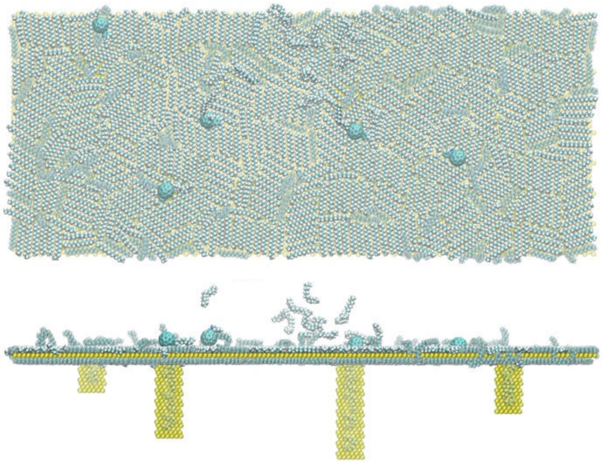

Figure 17: The short-length individual C_{60} hybrid adsorbed on $C_{14}H_{30}$-coated gold. It is modeled as a neutral molecule with a hydrogen atom added to cap the broken C-C linkage. Bulk *n*-$C_{14}H_{30}$ solvent molecules within 3 Å of gold are shown as transparent spheres.

Table 5: Computed interaction energies for short-length C_{60} hybrid molecule adsorption on spacer-coated gold (eV per broken molecule)

Interface	Molecule No.				
	1	2	3	4	5
Molecule / gold	-2.5 ± 0.0	-1.2 ± 0.1	-0.1 ± 0.0	-2.5 ± 0.1	-3.1 ± 0.0
Molecule / spacer layer	-0.7 ± 0.1	-0.9 ± 0.1	-0.5 ± 0.0	-0.9 ± 0.1	-0.7 ± 0.0
Molecule / bulk solvent	-1.9 ± 0.1	-1.9 ± 0.1	-2.4 ± 0.1	-1.6 ± 0.1	-1.6 ± 0.1
Net dimer stabilisation less SAM disruption penalty of +1.0 ± 0.1 eV/mol per C_{60}-Au contact					
	-4.1 ± 0.3 [one C_{60}-gold]	-4.0 ± 0.2	-3.0 ± 0.1	-4.0 ± 0.4 [one C_{60}-gold]	-4.4 ± 0.3 [one C_{60}-gold]

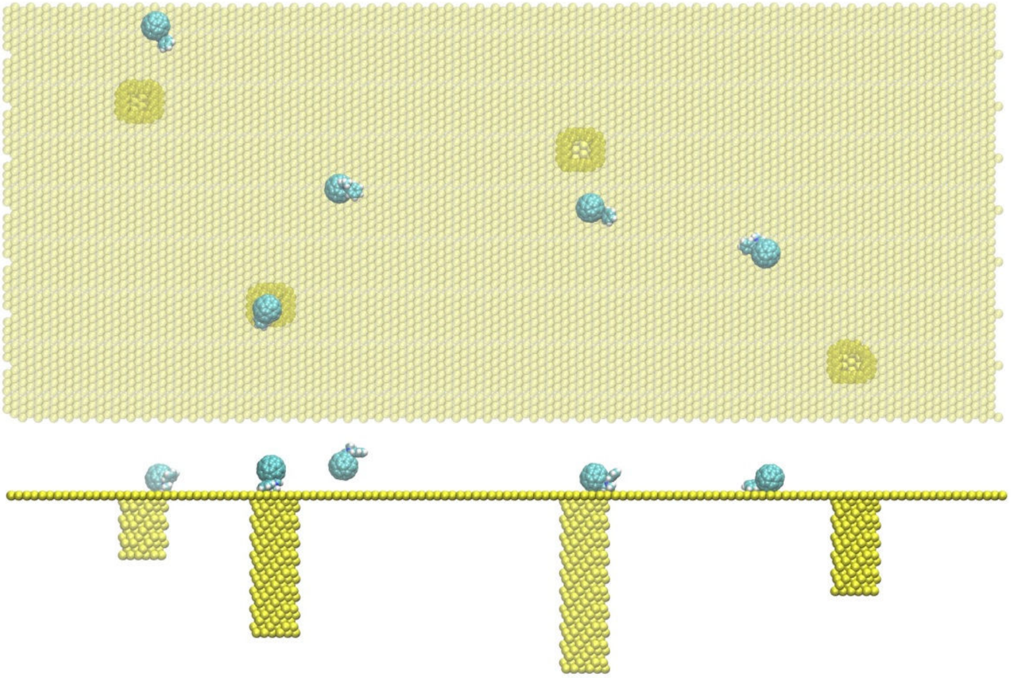

Figure 18: Adsorption modes of the short-length C_{60} hybrid with n-$C_{14}H_{30}$ molecules removed for clarity, showing the mix of on-spacer and on-gold adsorption modes present on the surface. Shown are top and side views of molecules adsorbed on the surface. Molecules are numbered 1-5 reading from left to right.

Calculated interaction energies (Table 5) indicate a small but significant benefit for direct gold contact for the short monomer. The preference for on-gold adsorption is at least 0.5 eV (Table 5) indicating that on-spacer species will be the minor population of the adsorbed short broken dimer fragments. Only one molecule is bound at a pore site, with the short tail interdigitated into the spacer layer. RMSF values for the short length C_{60} hybrid molecules (numbered 1-5) are 0.4 ± 0.2, 0.3 ± 0.2, 1.3 ± 0.5, 0.3 ± 0.1, and 0.2 ± 0.1 Å, where molecule no. 3 is the only molecule that adsorbs on top of the spacer layer. Hence is the short monomer is less mobile on average when directly contacted to gold than the intact dumbbell with one C_{60} bound to gold (RMSF = 0.5 ± 0.3 Å) due to the lack of competing (other) C_{60}-SAM interactions for the short molecule. By contrast, the short length C_{60} hybrid molecule is slightly more mobile on average on the spacer layer than the larger, intact dimer (RMSF = 1.1 ± 0.5 Å). The short length C_{60} hybrid molecules was also modelled as a radical anion (without a hydrogen atom added to cap the broken C-C linkage) to sample over the full range of possible monomeric species present in the experiments (as deduced from DFT data in section S8 above).

Table 6: Computed interaction energies for anionic short length C_{60} hybrid molecules adsorption on spacer-coated gold (eV per broken molecule)

Interface	Molecule No.				
	1	2	3	4	5
Molecule / gold	-2.5 ± 0.0	-0.2 ± 0.0	-0.1 ± 0.0	-2.5 ± 0.1	-2.9 ± 0.1
Molecule / spacer layer	-0.9 ± 0.1	-0.7 ± 0.1	-0.6 ± 0.0	-0.9 ± 0.1	-0.7 ± 0.0
Molecule / bulk solvent	-1.3 ± 0.1	-2.0 ± 0.1	-2.2 ± 0.1	-1.4 ± 0.1	-1.4 ± 0.1
Net dimer stabilisation less SAM disruption penalty of +1.0 (0.1) eV/mol per C_{60}-Au contact					
	-3.7 ± 0.3 [one C_{60}-gold]	-2.9 ± 0.2	-2.9 ± 0.1	-3.8 ± 0.4 [one C_{60}-gold]	-4.0 ± 0.3 [one C_{60}-gold]

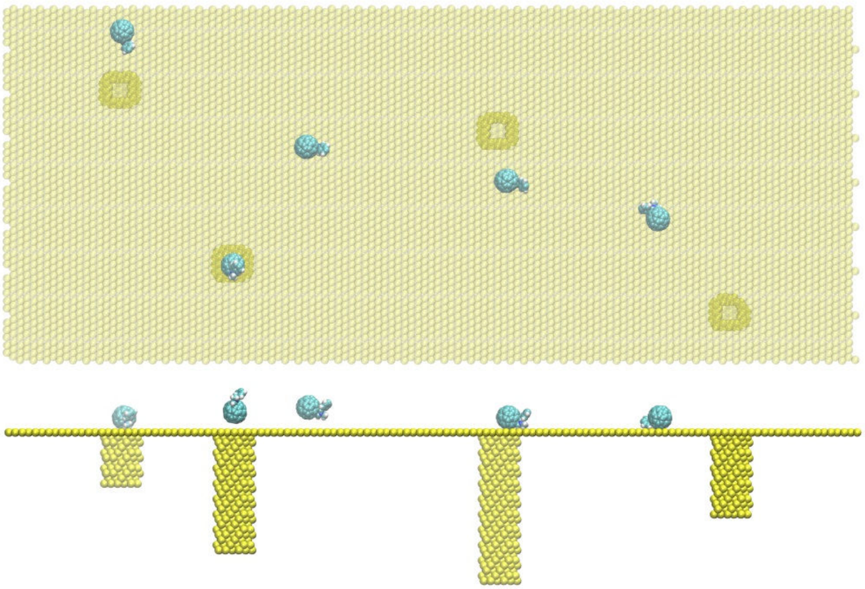

Figure 19: Adsorption modes of anionic short length C_{60} hybrid molecules with *n*-$C_{14}H_{30}$ molecules removed for clarity, showing the mix of on-spacer and on-gold adsorption modes present on the surface.

Structures for adsorption of the radical anion species were generally similar to those of the neutral species, as expected given the low polarity of the *n*-$C_{14}H_{30}$ spacer and solvent molecules. However, one point of interest from the simulations of the radical cation molecules is that the molecule bound on the pore (molecule #2) rotates its tail out of the spacer layer and removes the tail-gold contact that was present for the neutral species. It changes to an on-SAM adsorbed molecule as reflected in the energetic and dynamics data in Table 6. Every radical orients its charged phenyl ring up off the surface but the gold-C_{60} separation is not significantly increased relative to the neutral species. The minimum preference for on-gold adsorption is lowered to 0.3 eV, indicating a larger population of on-spacer adsorption modes for the radical compared with the neutral monomer. The RMSF values for the short radical molecules (numbered 1-5) are 0.5 ± 0.3, 1.1 ± 0.5, 1.5 ± 0.6, 0.5 ± 0.2, and 0.3 ± 0.1 Å. These values indicate that the charged tail slightly increases the fluctuations of the molecular positions as the tail seeks to relieve bad contacts with the non-polar *n*-$C_{14}H_{30}$ molecules, but the net mobility is not significantly increased. Note the slightly narrower and downwards-shifted RMSF distribution of the molecule adsorbed on the spacer near a pore site relative to the molecule adsorbed on the

spacer on the non-porous region (1.1 ± 0.5 Å *vs.* 1.5 ± 0.6 Å), illustrating a possible damping of molecular motion near pore sites.

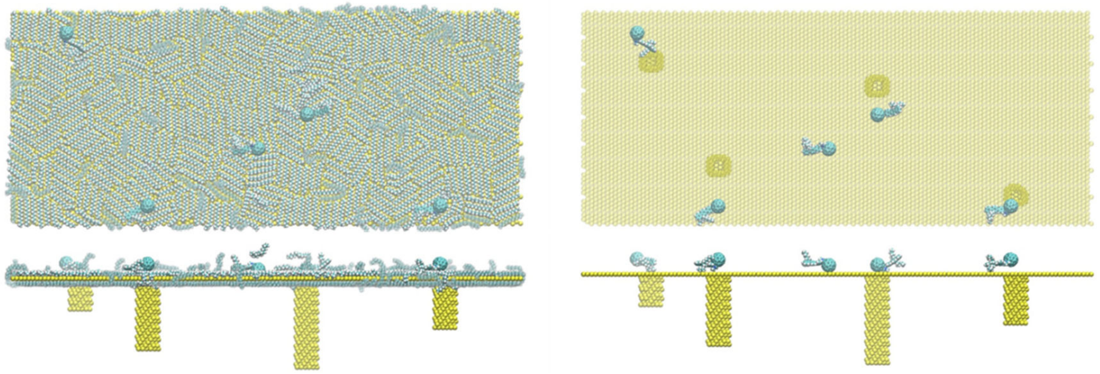

Figure 20: The long length C_{60} hybrid molecules adsorbed on spacer-coated gold, modelled as neutral molecules with a hydrogen atom added to cap the broken C-C linkage. (Left) Bulk solvent molecules within 3 Å of gold are shown as transparent spheres. (Right) All *n*-$C_{14}H_{30}$ molecules omitted for clarity.

The longer tail allows more extensive tail-gold contacts than for the short tailed species. The C_{60} group remains on top of the spacer layer for three of the five binding modes.

Table 7: Computed interaction energies for adsorption of the long length C_{60} hybrid molecules on spacer-coated gold (eV per broken molecule)

Interface	Molecule No.				
	1	2	3	4	5
Molecule / gold	-1.0 ± 0.0	-1.3 ± 0.1	-2.1 ± 0.0	-2.5 ± 0.0	-0.9 ± 0.1
Molecule / spacer layer	-1.2 ± 0.1	-1.3 ± 0.1	-1.5 ± 0.1	-1.4 ± 0.1	-1.3 ± 0.0
Molecule / bulk solvent	-3.5 ± 0.2	-2.8 ± 0.1	-3.1 ± 0.1	-3.0 ± 0.1	-3.0 ± 0.2
<u>Net dimer stabilisation less SAM disruption penalty of +1.0 (0.1) eV/mol per C_{60}-Au contact</u>					
	-5.7 ± 0.3	-5.4 ± 0.2	-5.7 ± 0.3 [one C_{60}-gold]	-5.9 ± 0.3	-5.2 ± 0.4 [one C_{60}-gold]

Computed RMSF values for the long-tailed monomers (numbered 1-5) are 0.7 ± 0.2, 0.4 ± 0.2, 0.5 ± 0.3, 0.3 ± 0.2, and 0.6 ± 0.3 Å.

Additional simulations of the long monomer modelled as a carbanion (charged, dehydrogenated terminal carbon atom produced as a result of C-C bond rupture in the dimer) showed very similar adsorption modes as those obtained for the neutral analogue. Computed interaction energies are given in Table 8.

Table 8: Computed interaction energies for adsorption of the radical anionic long length C_{60} hybrid molecules on spacer-coated gold (eV per broken molecule)

Interface	Molecule No.				
	1	2	3	4	5
Molecule / gold	-0.7 ± 0.0	-1.4 ± 0.1	-2.1 ± 0.0	-2.5 ± 0.1	-1.1 ± 0.0
Molecule / spacer layer	-1.1 ± 0.1	-1.5 ± 0.1	-1.7 ± 0.1	-1.4 ± 0.1	-1.4 ± 0.1
Molecule / bulk solvent	-4.1 ± 0.2	-3.2 ± 0.1	-3.2 ± 0.1	-3.4 ± 0.1	-3.1 ± 0.1
Net dimer stabilisation less SAM disruption penalty of +1.0 (0.1) eV/mol per C_{60}-Au contact					
	-5.9 ± 0.2	-6.1 ± 0.2	-6.0 ± 0.3 [one C_{60}-gold]	-6.3 ± 0.4	-5.6 ± 0.2 [one C_{60}-gold]

We find that the radical anion is slightly stabilised relative to the neutral species, reflecting the favourable contacts between the carbanion site and $C_{14}H_{30}$ hydrogens; this effect is minor for both the short and long monomers. Comparing short and long tailed monomers, the major difference is that on-gold and on-spacer adsorption modes are more distinguishable for the short fragment; for the long fragment the larger surface area means the molecule binds equally strongly irrespective of whether or not C_{60} makes a direct contact to gold. Computed RMSF values for the long broken anionic molecule (numbered 1-5) are 0.8 ± 0.4, 0.4 ± 0.1, 0.4 ± 0.2, 0.2 ± 0.1, and 0.4 ± 0.2 Å.

A final set of molecular dynamics simulations probed the structure, dynamics and energetics of adsorption of electrostically-bridged pairs of hybrid C_{60} molecules on the spacer-coated gold surface.

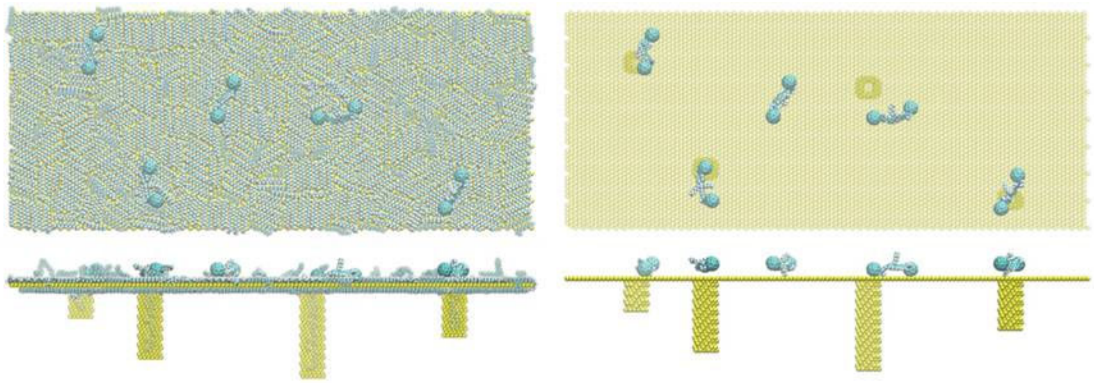

Figure 21: Adsorption of pairs of co-adsorbed charged C_{60} hybrids, in this case a cationic short fragment and an anionic long fragment. (Left) Bulk n-$C_{14}H_{30}$ solvent molecules within 3 Å of gold are shown as transparent spheres. (Right) All n-$C_{14}H_{30}$ molecules are omitted for clarity.

Table 9: Computed interaction energies for adsorption of electrostatically-bridged pairs of C_{60} hybrid molecules on spacer-coated gold (eV per pair)

Interface	Molecule No. 1	2	3	4	5
Molecule / gold	-2.5 ± 0.1	-0.4 ± 0.1	-0.7 ± 0.0	-4.5 ± 0.1	-0.9 ± 0.1
Molecule / spacer layer	-2.2 ± 0.1	-2.1 ± 0.1	-2.2 ± 0.1	-2.1 ± 0.1	-1.8 ± 0.1
Molecule / bulk solvent	-4.6 ± 0.2	-5.5 ± 0.2	-5.3 ± 0.2	-4.2 ± 0.1	-5.7 ± 0.2
Net dimer stabilisation less SAM disruption penalty of +1.0 (0.1) eV/mol per C_{60}-Au contact					
	-8.3 ± 0.5	-8.0 ± 0.3	-8.2 ± 0.3	-8.8 ± 0.4	-8.4 ± 0.5
	[one C_{60}-gold contact]			[two C_{60}-gold contacts]	

The simulations show that a multitude of binding modes are available for these weakly-bound, temporarily-associated, electrostatically bridged pairs, with various combinations of tail and C_{60} anchor groups adsorbed on the spacer layer and directly contacted to gold. The molecule can adsorb entirely on the spacer layer without any long-lived significant

contacts with gold (the second adsorption mode, reading from left to right in Fig. 21), along with the variety of other approximately iso-energetic binding modes available in the room temperature $C_{14}H_{30}$_liquid/molecule/$C_{14}H_{30}$_spacer/gold cell. The electrostatic bridge between the cation and anion has a computed binding energy of -4.0 ± 0.1 eV calculated over all atoms in the terminal charged ring in both ions. This is comparable to the C-C bond dissociation energy of -3.9 eV measured in biphenyl.[12] Collisions with *n*-$C_{14}H_{30}$ spacer and solvent molecules do not break the electrostatic bridge during the few-nanosecond timescale of the simulations but will give a distribution of bridged and dissociated states on experimental timescales due to the reversible, non-covalent nature of the contact between the cation and anion pair. RMSF values for the bridged pairs of hybrid C_{60} molecules are 0.8 ± 0.2, 0.6 ± 0.2, 0.9 ± 0.3, 0.3 ± 0.2, and 1.0 ± 0.5 Å. Hence, fluctuations in molecular position (prior to dissociation) are similar to those calculated for the regular dimer. Almost identical structures and data were obtained for an alternative pair of co-adsorbed hybrids; an anionic short fragment and a cationic long fragment.

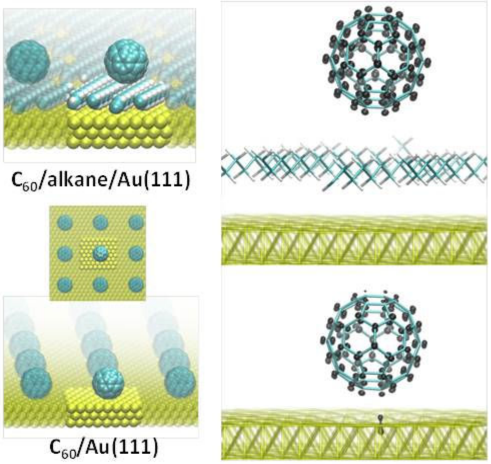

Figure 22: Computed electron densities in the energy range $E_f \rightarrow (E_f - 1.0)$ eV. The two models for C_{60} adsorption, on alkane-coated gold (top panel) and bare gold (bottom panel), are shown in the left hand panels with atoms drawn as space-filling spheres and neighboring periodic images shown as transparent spheres. Carbon atoms are coloured cyan, hydrogen atoms are coloured white and gold atoms are coloured yellow. Computed electron densities are plotted in the right hand panels using a calculated charge isovalue of 0.1 e/Å^3, showing the predominance of C_{60} 2p orbitals to the HOMO region. Atoms

are shown in stick representation with charge density coloured black. The C_{60} electronic structure is slightly perturbed by gold when the alkane layer is not present, with a Au $5d_z^2$ orbital making a minor contribution to the near-Fermi band. The density functional theory (DFT) electronic structure calculations were performed using the VASP program [13]. The periodic surface models were described using periodic plane wave DFT with the GGA-PBE functional [14], projector augmented wave (PAW) pseudopotentials [15] with a plane wave cut-off of 400 eV and a vacuum spacing of approximately 3 nm in the direction normal to the gold surface. The molecule-surface complexes were calculated using periodic boundary conditions. Molecule-surface binding energies were converged to below 100 meV using a 441 k-point grid. Chemisorption energies (ΔE_c) were calculated by comparing the electronic energies of the complex with the isolated metal and molecule.

References

1. Long, Y. et al. Electrospun Nanofibrous Film Doped with a Conjugated Polymer for DNT Fluorescence Sensor. *Macromolecules* **42**, 6501-6509 (2009).
2. Maggini, M., Scorrano, G. & Prato, M. Addition of azomethine ylides to C60: synthesis, characterization, and functionalization of fullerene pyrrolidines. *Journal of the American Chemical Society* **115**, 9798-9799 (1993).
3. Prato, M. et al. Optical Characterization of Thiolate Self-Assembled Monolayers on Au(111). *The Journal of Physical Chemistry C* **112**, 3899-3906 (2008).
4. Humphrey, W., Dalke, A. & Schulten, K. VMD: Visual molecular dynamics. *Journal of Molecular Graphics* **14**, 33-38 (1996).
5. Lortscher, E., Widmer, D. & Gotsmann, B. Next-generation nanotechnology laboratories with simultaneous reduction of all relevant disturbances. *Nanoscale* **5**, 10542-10549 (2013).
6. Becke, A.D. Density-functional theory, Thermochemistry 3. Role of exact exchange. *J. Chem. Phys* **98** (1993).
7. Stephens, P.J., Devlin, F.J., Chabalowski, C.F. & Frisch, M.J. Ab Initio Calculation of Vibrational Absorption and Circular Dichroism Spectra Using Density Functional Force Fields. *The Journal of Physical Chemistry* **98**, 11623-11627 (1994).
8. Hanwell, M. et al. Avogadro: an advanced semantic chemical editor, visualization, and analysis platform. *Journal of Cheminformatics* **4**, 17 (2012).
9. MacKerell, A.D. et al. All-Atom Empirical Potential for Molecular Modeling and Dynamics Studies of Proteins. *The Journal of Physical Chemistry B* **102**, 3586-3616 (1998).
10. Heinz, H., Vaia, R.A., Farmer, B.L. & Naik, R.R. Accurate Simulation of Surfaces and Interfaces of Face-Centered Cubic Metals Using 12−6 and 9−6

Lennard-Jones Potentials. *The Journal of Physical Chemistry C* **112**, 17281-17290 (2008).
11. Phillips, J.C. et al. Scalable molecular dynamics with NAMD. *Journal of Computational Chemistry* **26**, 1781-1802 (2005).
12. S. Fliszar (2008) in: Atomic Charges, Bond Properties, and Molecular Energies. John Wiley & Sons, p. 144.
13. Kresse, G. & Hafner, J. Abinitio Molecular-Dynamics for Liquid-Metals. *Phys Rev B* **47**, 558-561 (1993).
14. Perdew, J.P., Burke, K. & Ernzerhof, M. Generalized gradient approximation made simple (vol 77, pg 3865, 1996). *Phys Rev Lett* **78**, 1396-1396 (1997).
15. Blochl, P.E. Projector Augmented-Wave Method. *Phys Rev B* **50**, 17953-17979 (1994).
